# Supplementary figures and images for: Protein nanobarcodes enable single-step multiplexed fluorescence imaging
Source: PLoS Biol. 2023 Dec 11;21(12):e3002427. doi: 10.1371/journal.pbio.3002427 (PMC10735187; doi:10.1371/journal.pbio.3002427)

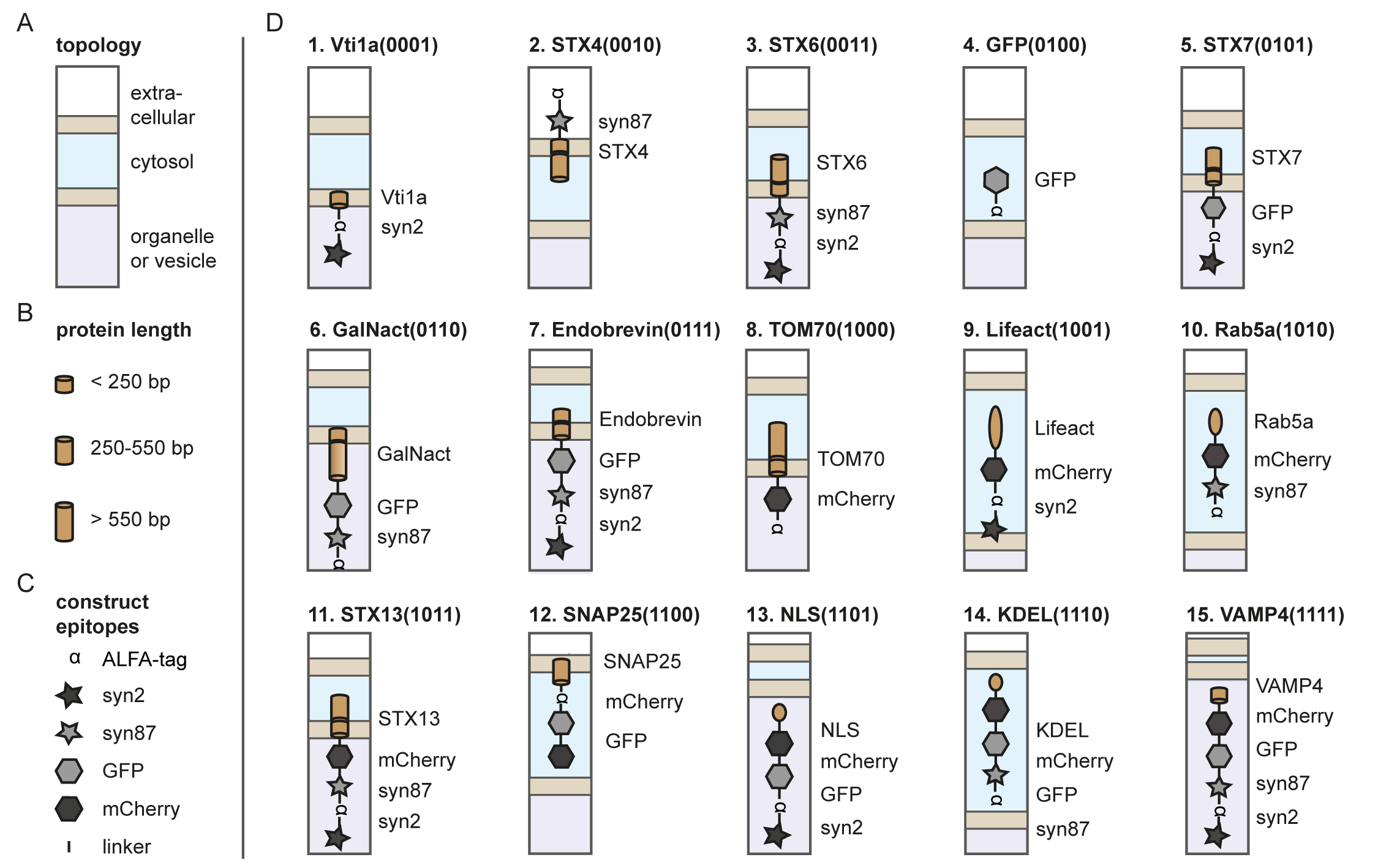

Supplement: S1 Fig — (A-C) Legends for expected topology (A), protein length (B), and construct epitopes (C). (D) Protein topology schemes for the 15 constructs used. Below is a list with detailed information about the respective topology scheme of each construct depicted in (B). Uniprot accession numbers (acc.nr.) are available under https://www.uniprot.org/uniprot/. Sequences of all constructs are listed in “plasmid_sequence_information.xlsx” stored in “Plasmid_design.zip” available from http://dx.doi.org/10.17169/refubium-40101. No protein, used for background signals. (TIF) [file pbio.3002427.s001.tif]

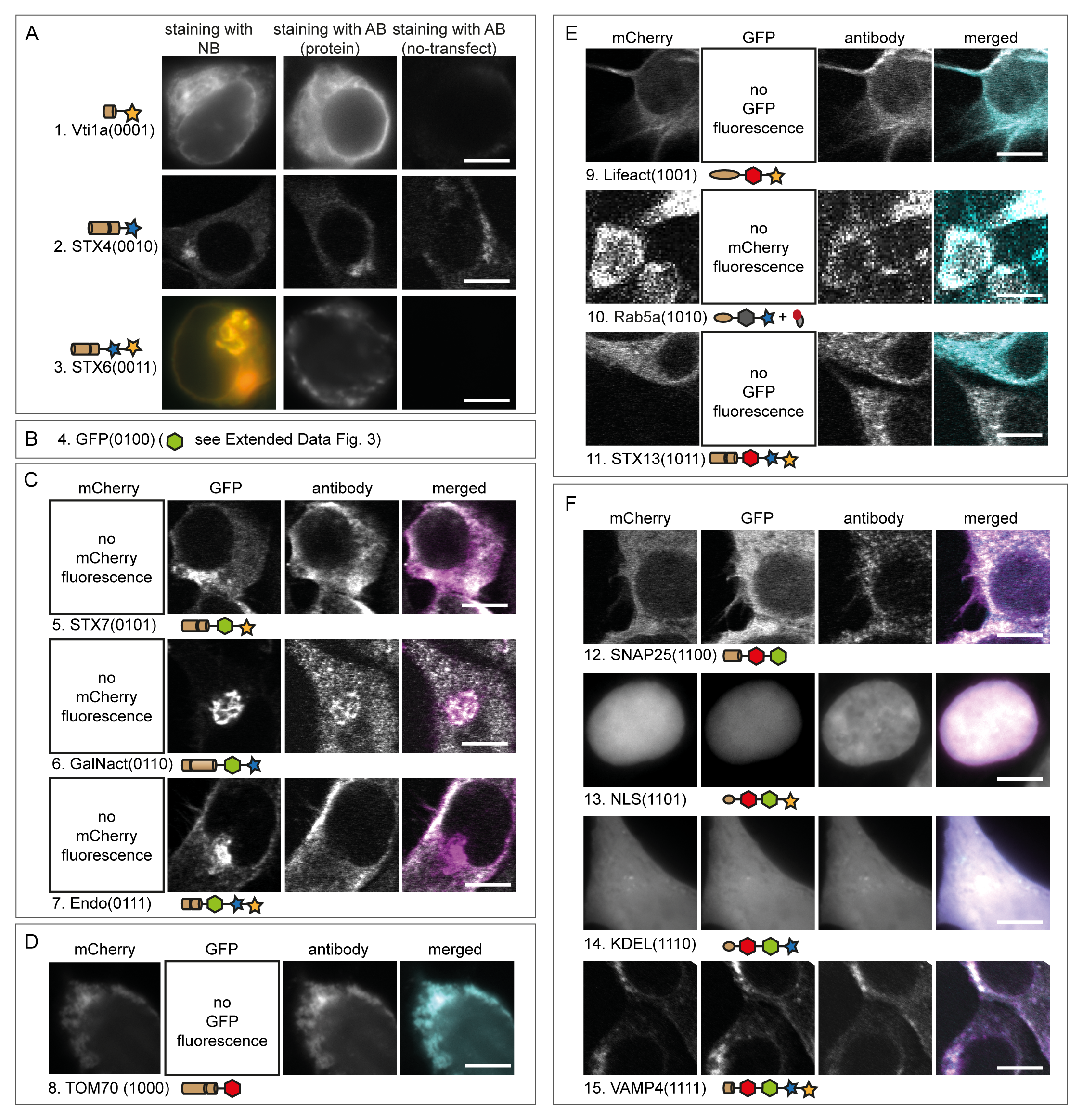

Supplement: S2 Fig — We validated the correct nanobarcoding and expression of the protein constructs by simultaneous visualization of the nanobarcodes and their respective endogenous epitope counterparts. The nanobarcodes were visualized by imaging their GFP or mCherry fluorescence (relying on constructs lacking the Y/L mutations of the chromophores), or by nanobody stainings, for barcodes lacking GFP or mCherry. We immunostained the respective proteins of interest with antibodies directed against protein-specific epitopes. (A) All protein constructs lacking mCherry or GFP fluorescence. (B, C) Protein constructs with GFP fluorescence. These proteins exhibit a strong localization to the perinuclear area, where antibodies penetrate more poorly than nanobodies [69]. See S3 and S4 Figs for nanobody staining of the nanobarcode epitopes. (D, E) All protein constructs having a fluorescent mCherry epitope. (F) All protein constructs having mCherry and GFP fluorescence. To visualize the target protein component of the nanobarcoded proteins, Cy5-coupled secondary antibodies were used. Scale bars: 10 μm. (TIF) [file pbio.3002427.s002.tif]

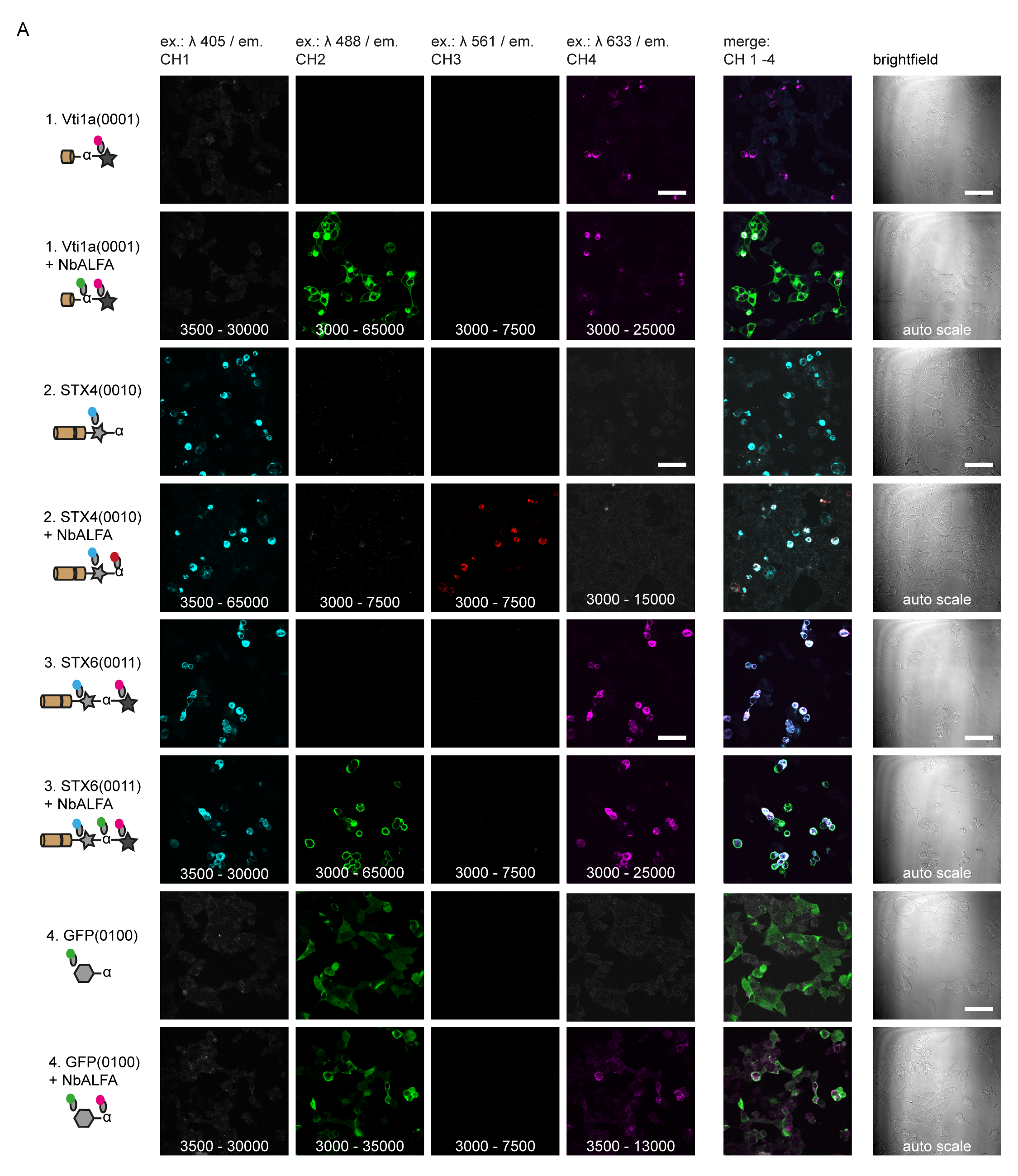

Supplement: S3 Fig — (A-D) Nanobody-based identification of the 4 genetically encoded nanobarcode epitopes mCherry(Y71L), GFP(Y66L), syn87, and syn2 and the ALFA-tag epitope by their corresponding nanobodies NbRFP, NbEGFP, NbSyn87, NbSyn2, and NbALFA. Scaling was optimized for each protein. (D) VAMP4(1111) example (all epitopes present) and negative control condition: mock transfection (no DNA, no epitopes present) using same intensity scale. (E) As in (D), now with upscale intensities. Scale bar: 50 μm. (TIFF) [file pbio.3002427.s003.tiff]

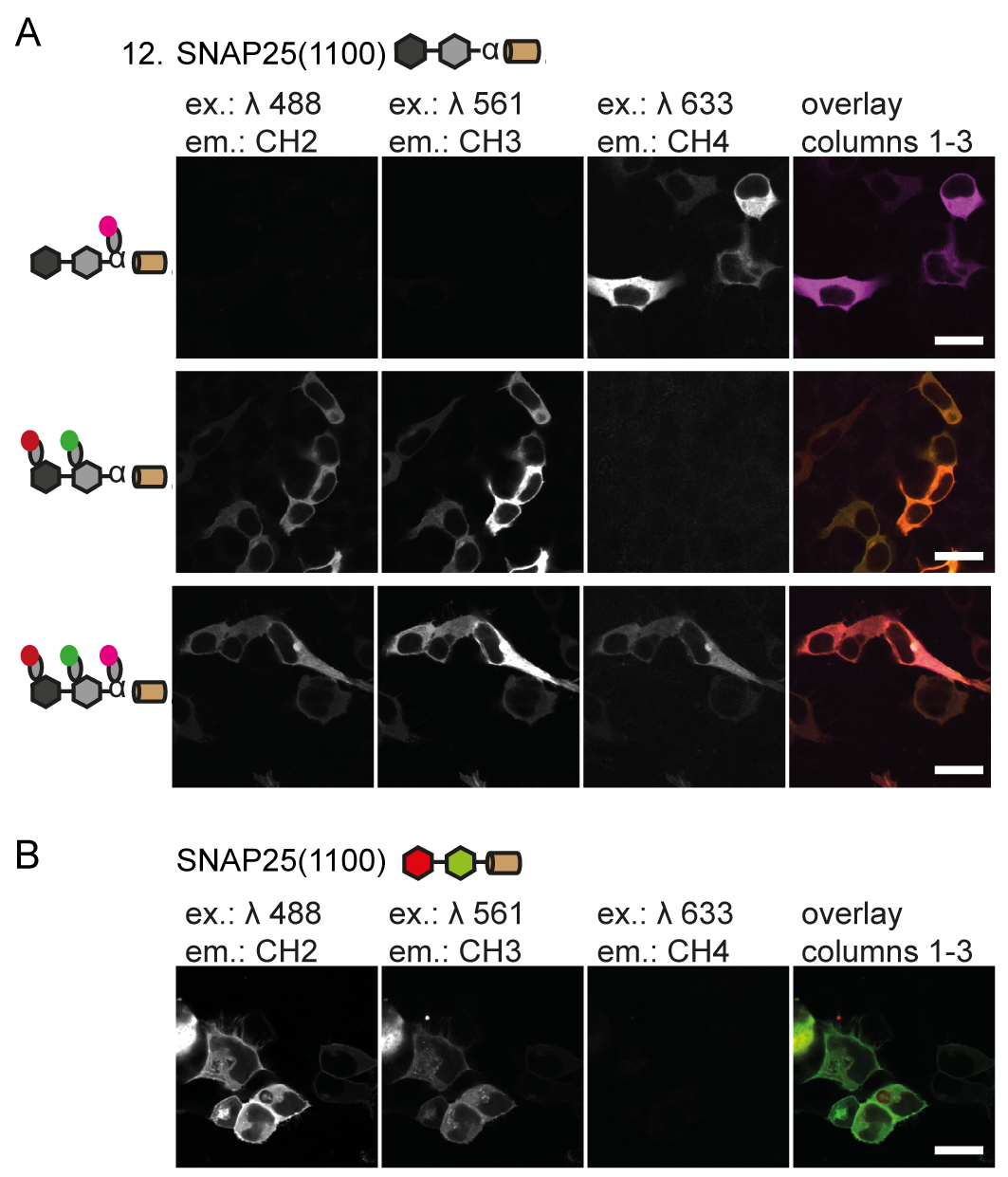

Supplement: S4 Fig — (A) Nonfluorescent epitopes of SNAP25(1100) are recognized successfully by the corresponding nanobodies, independent of the number of nanobodies used. The SNAP25(1100) construct is successfully stained when using the NbALFA only (first row), when using the anti-GFP and anti-RFP nanobodies (second row) or when using the anti-GFP, anti-RFP, and anti-ALFA nanobodies (third row). The corresponding nanobarcode epitopes are detected, which indicates that there is no substantial steric hindrance between the nanobodies (which is expected due to the small size of the nanobodies). The morphological features of the cells are similar to cells transfected with a SNAP25 construct containing fluorescent epitopes (B), which indicates that these constructs are comparable and, therefore, suitable for our investigations, as shown in Figs 3 and 4. Scale bars: 30 μm. (TIF) [file pbio.3002427.s004.tif]

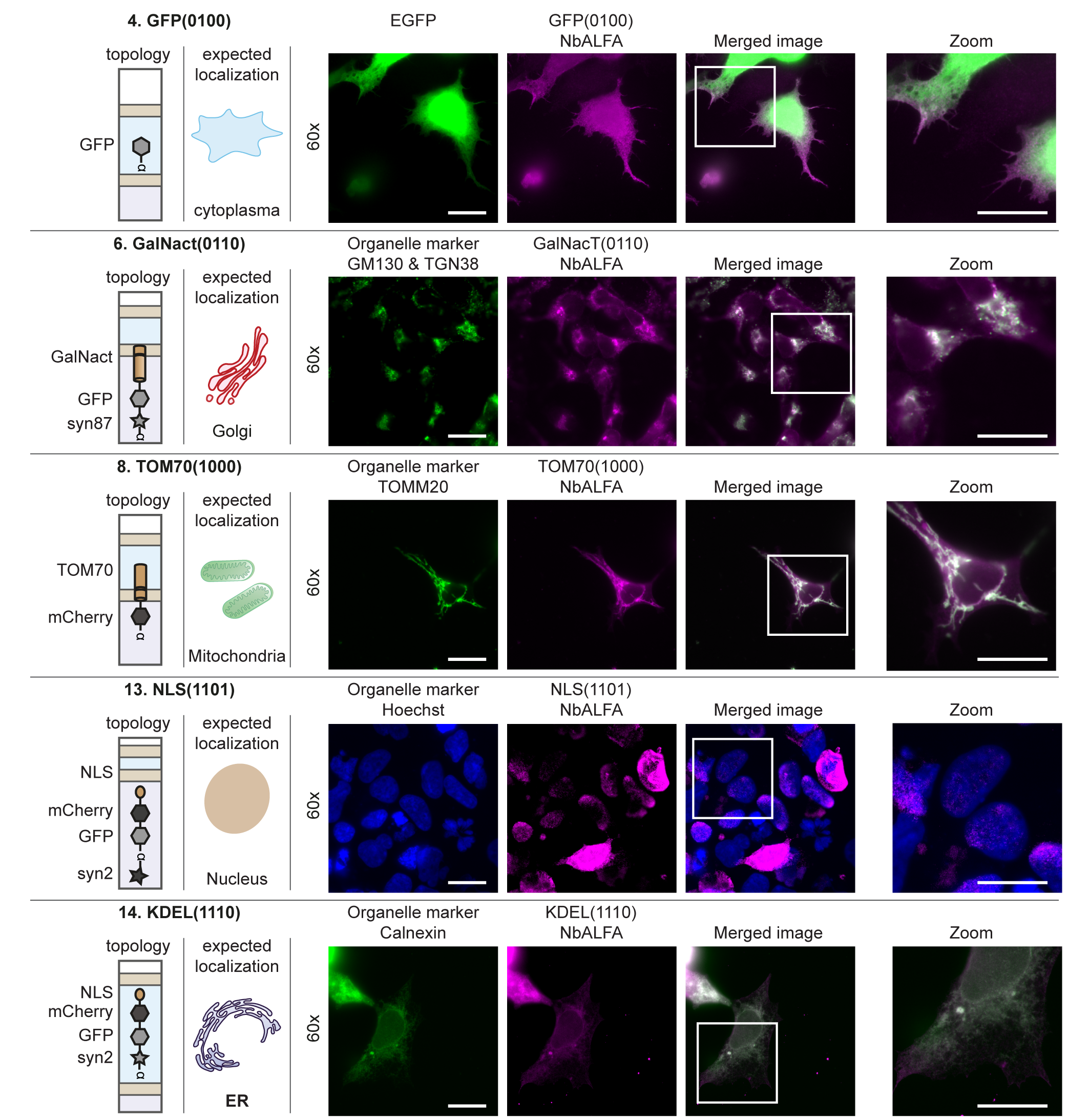

Supplement: S5 Fig — The proteins indicated in the left-most column are markers for specific compartments, indicated in the next column. The colocalization of these proteins and specific compartment markers is then indicated in the fluorescence images. Scale bars: 20 μm. For quantification, see S17 Fig, below. (TIF) [file pbio.3002427.s005.tif]

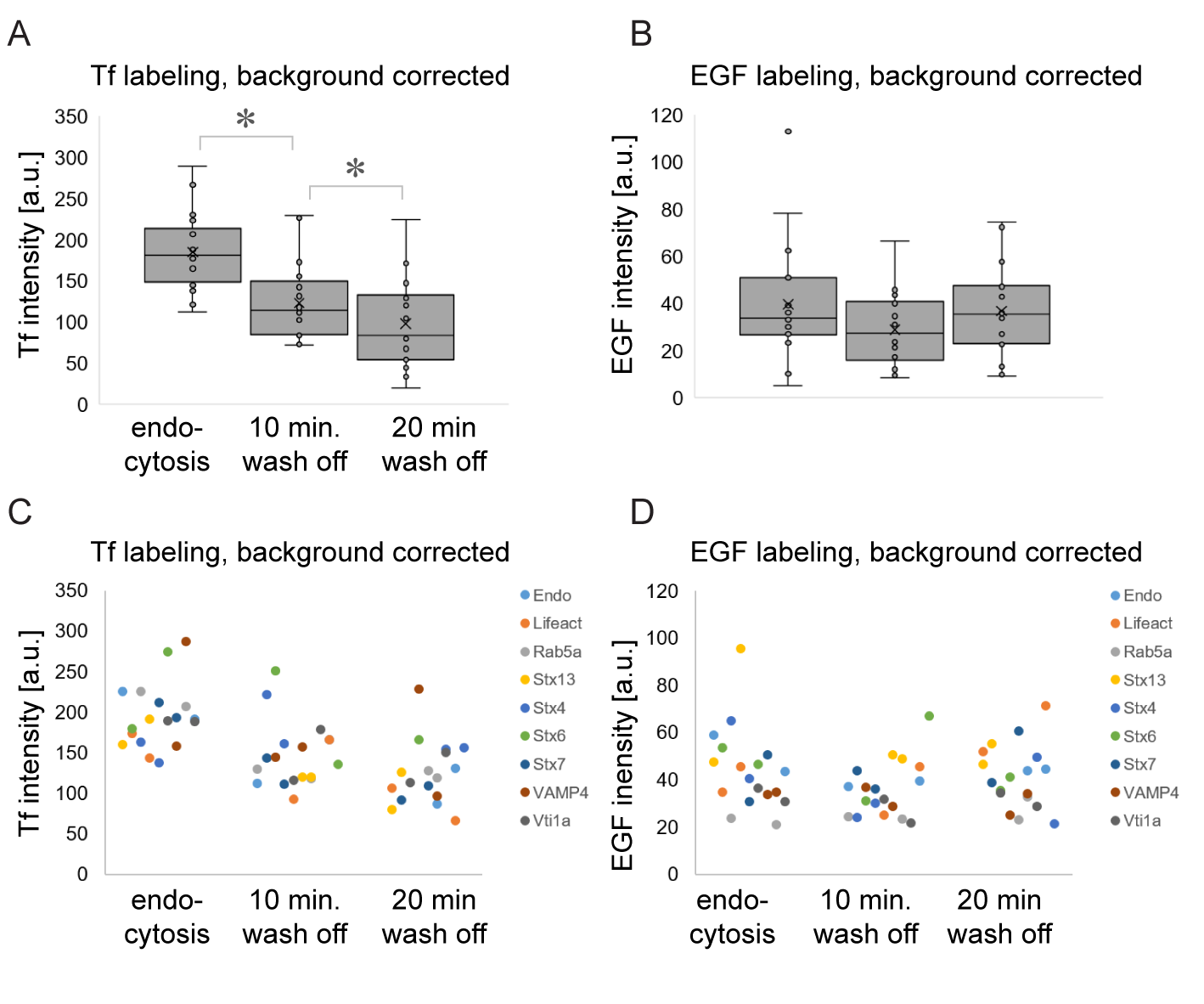

Supplement: S6 Fig — Cells expressing different nanobarcoded proteins were pulsed with transferrin conjugated to Alexa488 and with EGF conjugated to Alexa647, for 10 minutes, allowing the cells to endocytose these ligands. Afterwards, they were immediately fixed or were chased (washed off) in a minimal buffer at 37°C, for 10 or 20 minutes. Finally, all cells were fixed and immunolabeled for the ALFA tag, to identify the nanobarcoded proteins. (A, B) The behavior of transferrin and EGF, respectively. Transferrin recycles, as expected, being released during the chase period (Kruskal–Wallis test followed by Tukey post hoc test, p < 0.006 for endocytosis vs. 10- or 20-minute wash-off). No changes were seen for EGF, as expected (Kruskal–Wallis test, not significant). N = 17–18 independent experiments. (C, D) Same data as above, but indicating the nature of the nanobarcoded protein in each of the independent experiments. The data underlying this Figure can be found in the following Sheets of the “S1 Data file: “Tf_SFig 6A,” “EGF_SFig 6B,” “Tf_SFig 6C,” and “EGF_SFig 6D.” The S1 Data file is available from http://dx.doi.org/10.17169/refubium-40101. (TIF) [file pbio.3002427.s006.tif]

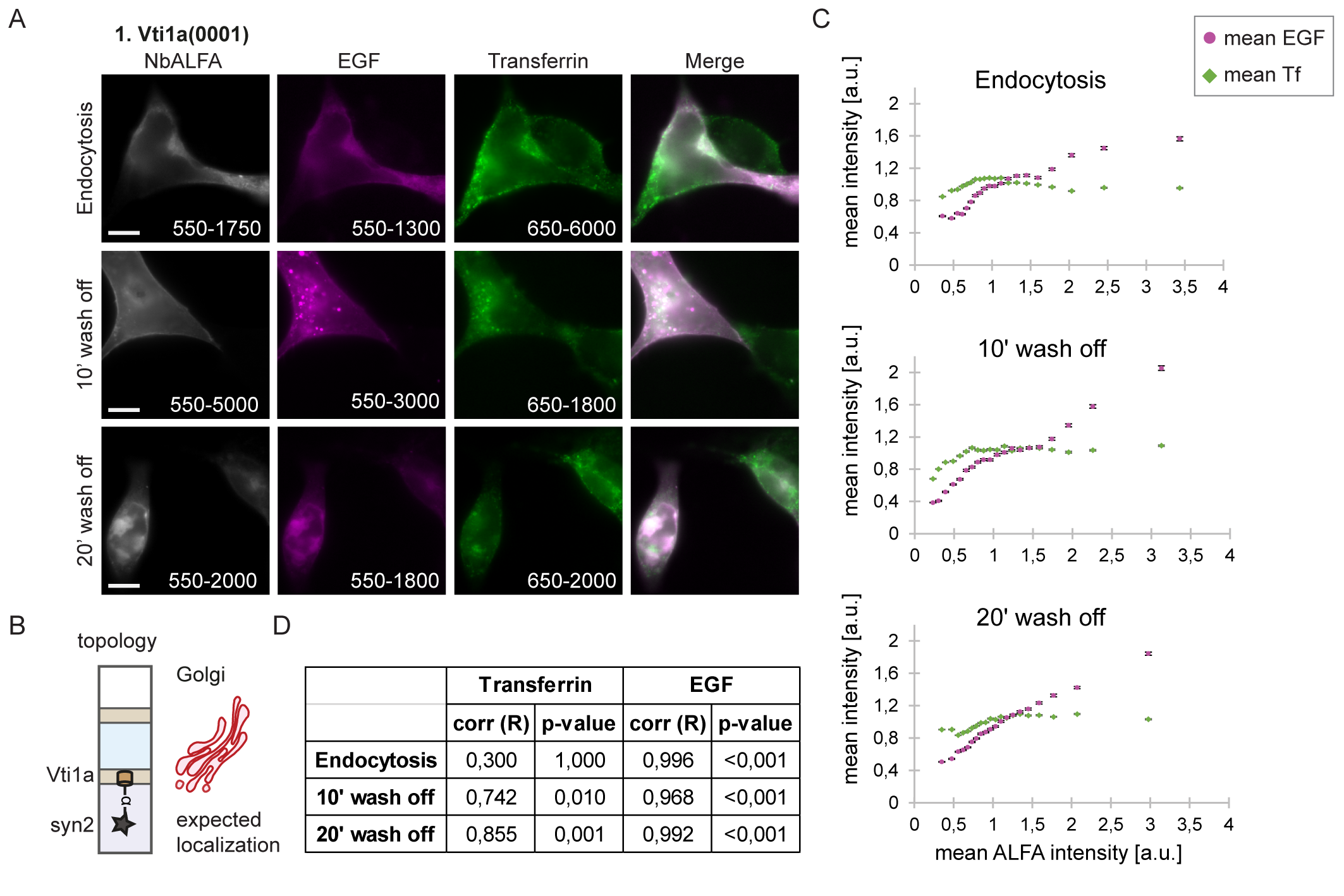

Supplement: S7 Fig — (A) Visualization of transferrin-Alexa488 (green) and EGF-Alexa647 (magenta), as well as the transfected protein, visualized with the ALFA nanobody (NbALFA) conjugated to AZdye568 (white). The 3 rows show the 10-minute pulse with the ligands (endocytosis), followed by the 10- and 20-minute chase (wash-off). To enable optimal visualization, the images are scaled differently, with the image scaling indicated in all panels. Scale bars: 20 μm. (B) The nanobarcoding scheme and the expected localization of the protein. (C) The NbALFA fluorescence intensity is plotted against the transferrin (green) and EGF (magenta) intensity, for all signals measured in 2 independent experiments, for all conditions. All intensities were normalized to the medians of the distributions and were then grouped in 20 bins of ALFA intensity, each containing similar numbers of values. The mean and SEM of each bin in the respective channels are plotted. The data underlying this Figure can be found in the S1 Data file, Sheet “SFig 7C_Vti1a,” available from http://dx.doi.org/10.17169/refubium-40101. (D) The Pearson’s correlation coefficients for the distributions from panel C are shown, with the p-values corrected for multiple testing using a Bonferroni correction. (TIF) [file pbio.3002427.s007.tif]

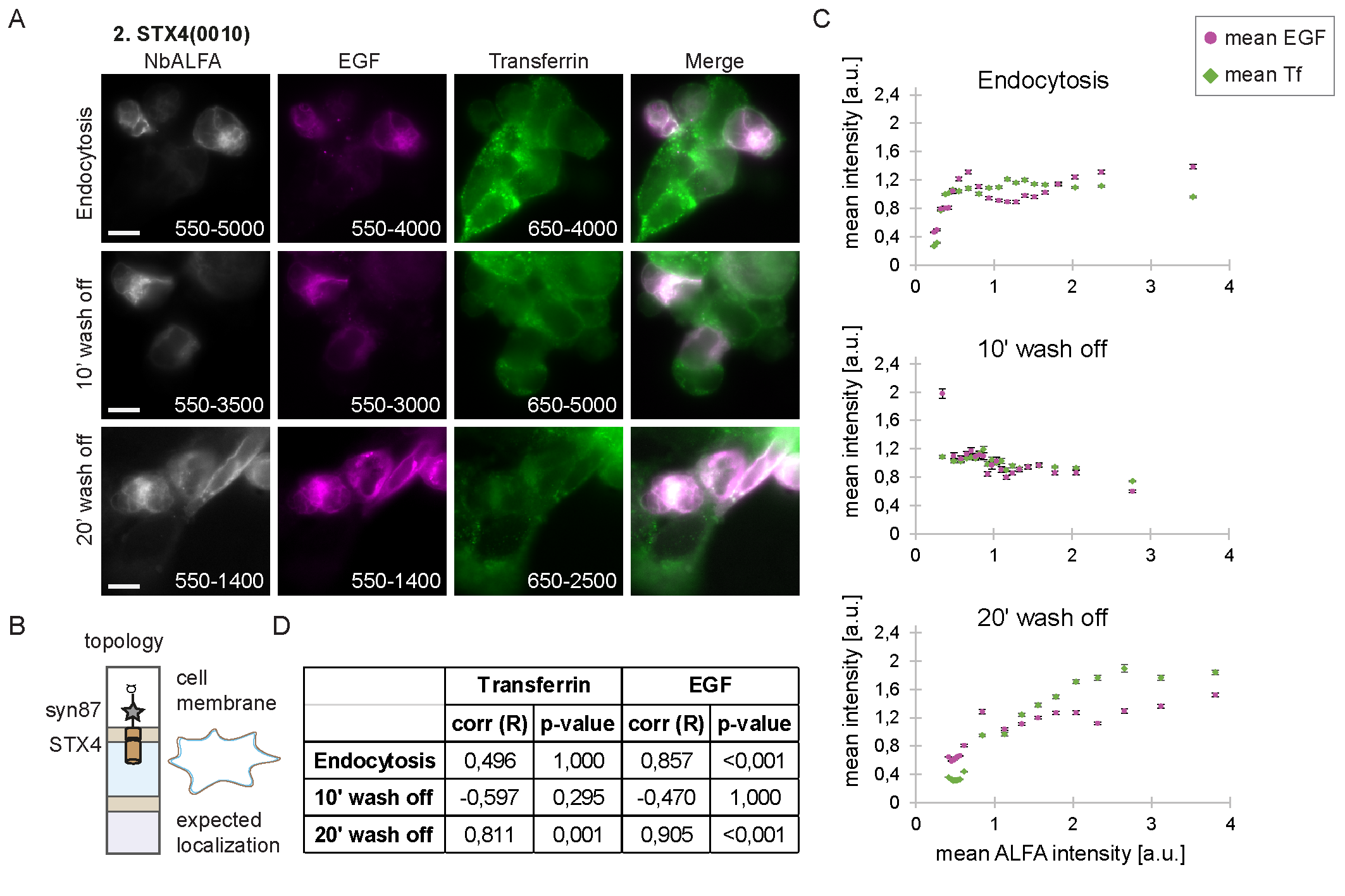

Supplement: S8 Fig — (A) Visualization of transferrin-Alexa488 (green) and EGF-Alexa647 (magenta), as well as the transfected protein, visualized with the ALFA nanobody (NbALFA) conjugated to AZdye568 (white). The 3 rows show the 10-minute pulse with the ligands (endocytosis), followed by the 10- and 20-minute chase (wash-off). To enable optimal visualization, the images are scaled differently, with the image scaling indicated in all panels. Scale bar: 20 μm. (B) The nanobarcoding scheme and the expected localization of the protein. (C) The NbALFA fluorescence intensity is plotted against the transferrin (green) and EGF (magenta) intensity, for all signals measured in 2 independent experiments, for all conditions. All intensities were normalized to the medians of the distributions and were then grouped in 20 bins of ALFA intensity, each containing similar numbers of values. The mean and SEM of each bin in the respective channels are plotted. The data underlying this Figure can be found in the S1 Data file, Sheet “SFig 8C_STX4,” available from http://dx.doi.org/10.17169/refubium-40101. (D) The Pearson’s correlation coefficients for the distributions from panel C are shown, with the p-values corrected for multiple testing using a Bonferroni correction. (PNG) [file pbio.3002427.s008.png]

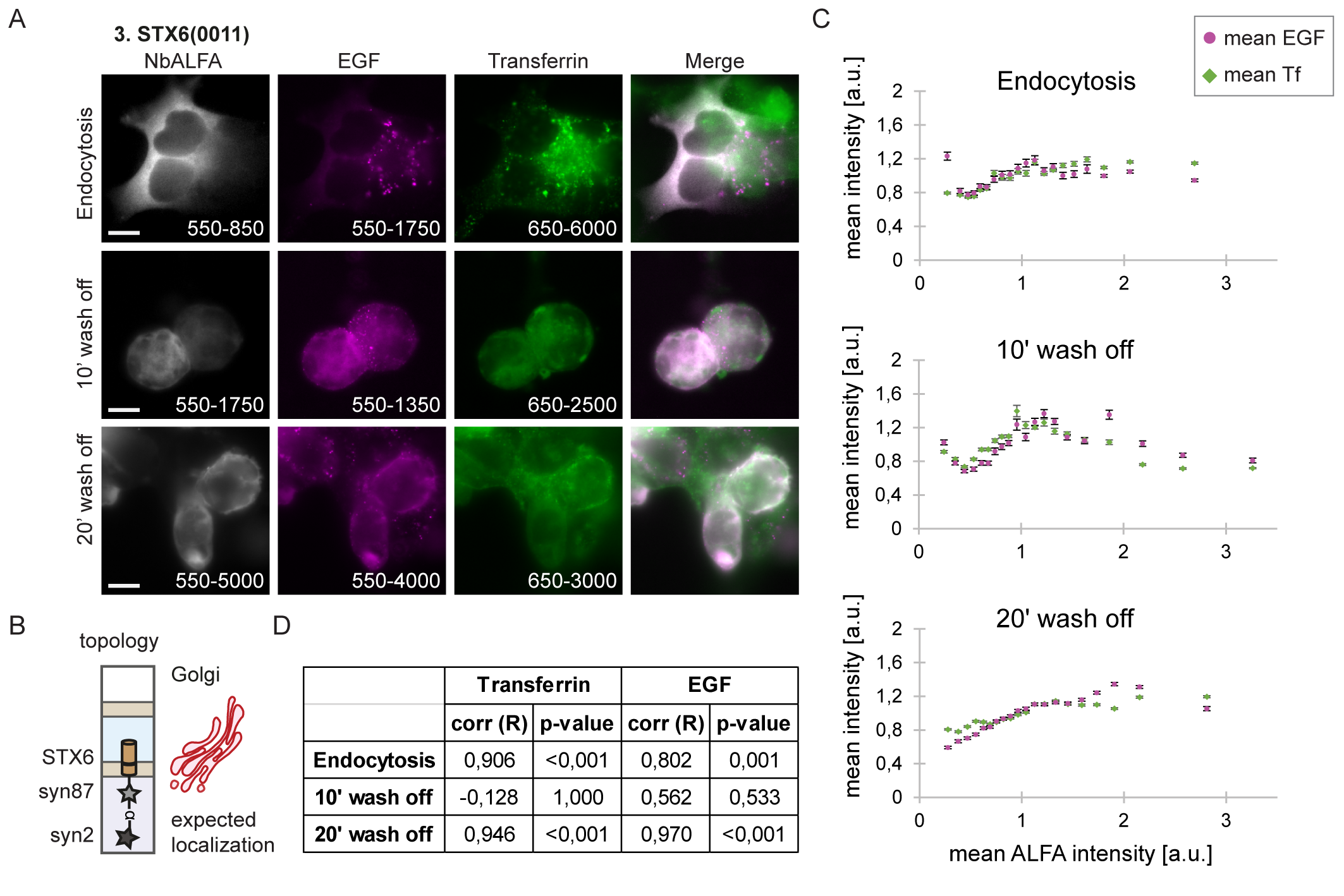

Supplement: S9 Fig — (A) Visualization of transferrin-Alexa488 (green) and EGF-Alexa647 (magenta), as well as the transfected protein, visualized with the ALFA nanobody (NbALFA) conjugated to AZdye568 (white). The 3 rows show the 10-minute pulse with the ligands (endocytosis), followed by the 10- and 20-minute chase (wash-off). To enable optimal visualization, the images are scaled differently, with the image scaling indicated in all panels. Scale bar: 20 μm. (B) The nanobarcoding scheme and the expected localization of the protein. (C) The NbALFA fluorescence intensity is plotted against the transferrin (green) and EGF (magenta) intensity, for all signals measured in 2 independent experiments, for all conditions. All intensities were normalized to the medians of the distributions and were then grouped in 20 bins of ALFA intensity, each containing similar numbers of values. The mean and SEM of each bin in the respective channels are plotted. The data underlying this Figure can be found in the S1 Data file, Sheet “SFig 9C_STX6,” available from http://dx.doi.org/10.17169/refubium-40101. (D) The Pearson’s correlation coefficients for the distributions from panel C are shown, with the p-values corrected for multiple testing using a Bonferroni correction. (TIF) [file pbio.3002427.s009.tif]

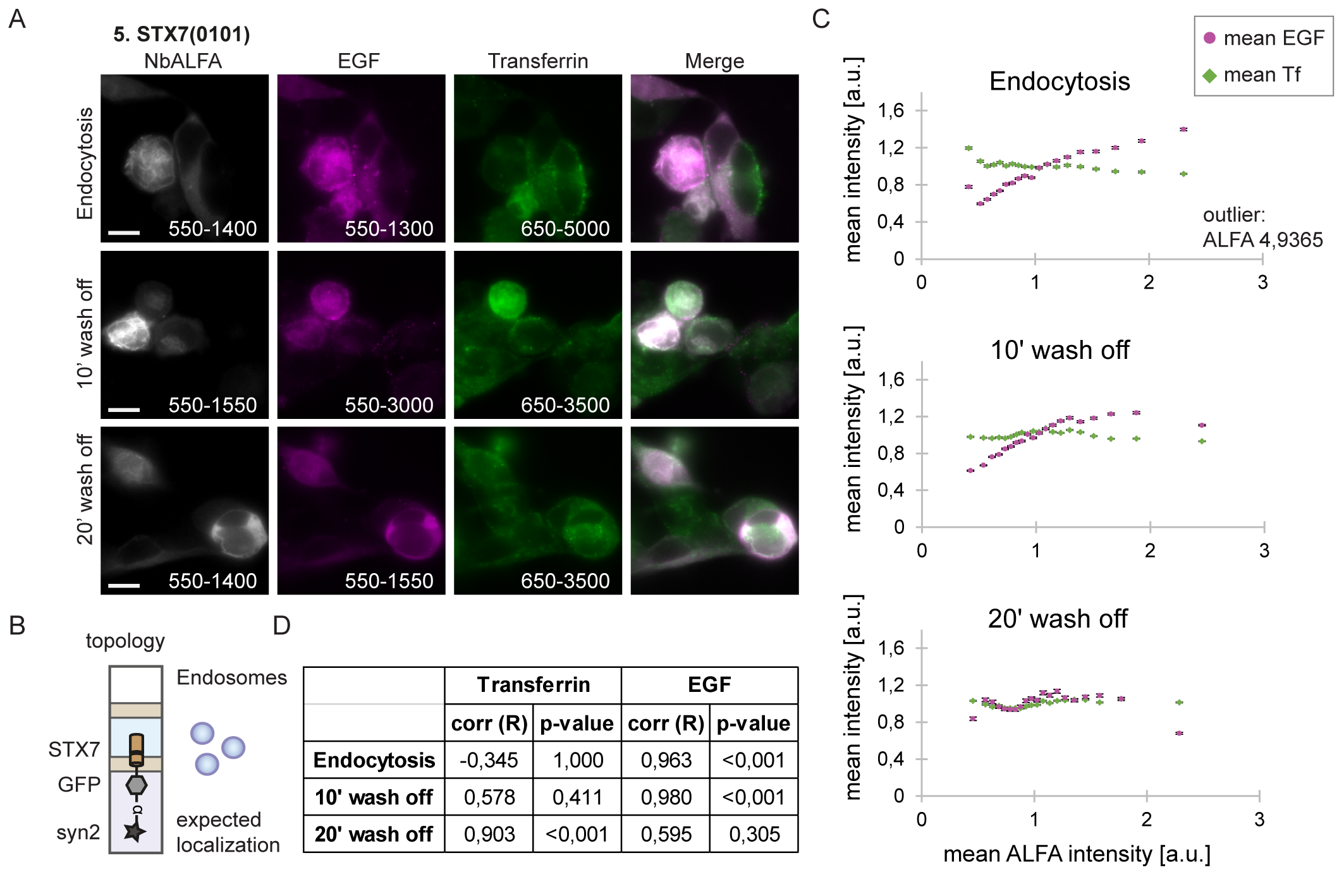

Supplement: S10 Fig — (A) Visualization of transferrin-Alexa488 (green) and EGF-Alexa647 (magenta), as well as the transfected protein, visualized with the ALFA nanobody (NbALFA) conjugated to AZdye568 (white). The 3 rows show the 10-minute pulse with the ligands (endocytosis), followed by the 10- and 20-minute chase (wash-off). To enable optimal visualization, the images are scaled differently, with the image scaling indicated in all panels. Scale bar: 20 μm. (B) The nanobarcoding scheme and the expected localization of the protein. (C) The NbALFA fluorescence intensity is plotted against the transferrin (green) and EGF (magenta) intensity, for all signals measured in 2 independent experiments, for all conditions. All intensities were normalized to the medians of the distributions and were then grouped in 20 bins of ALFA intensity, each containing similar numbers of values. The mean and SEM of each bin in the respective channels are plotted. The data underlying this Figure can be found in the S1 Data file, Sheet “SFig 10C_STX7,” available from http://dx.doi.org/10.17169/refubium-40101. (D) The Pearson’s correlation coefficients for the distributions from panel C are shown, with the p-values corrected for multiple testing using a Bonferroni correction. (TIF) [file pbio.3002427.s010.tif]

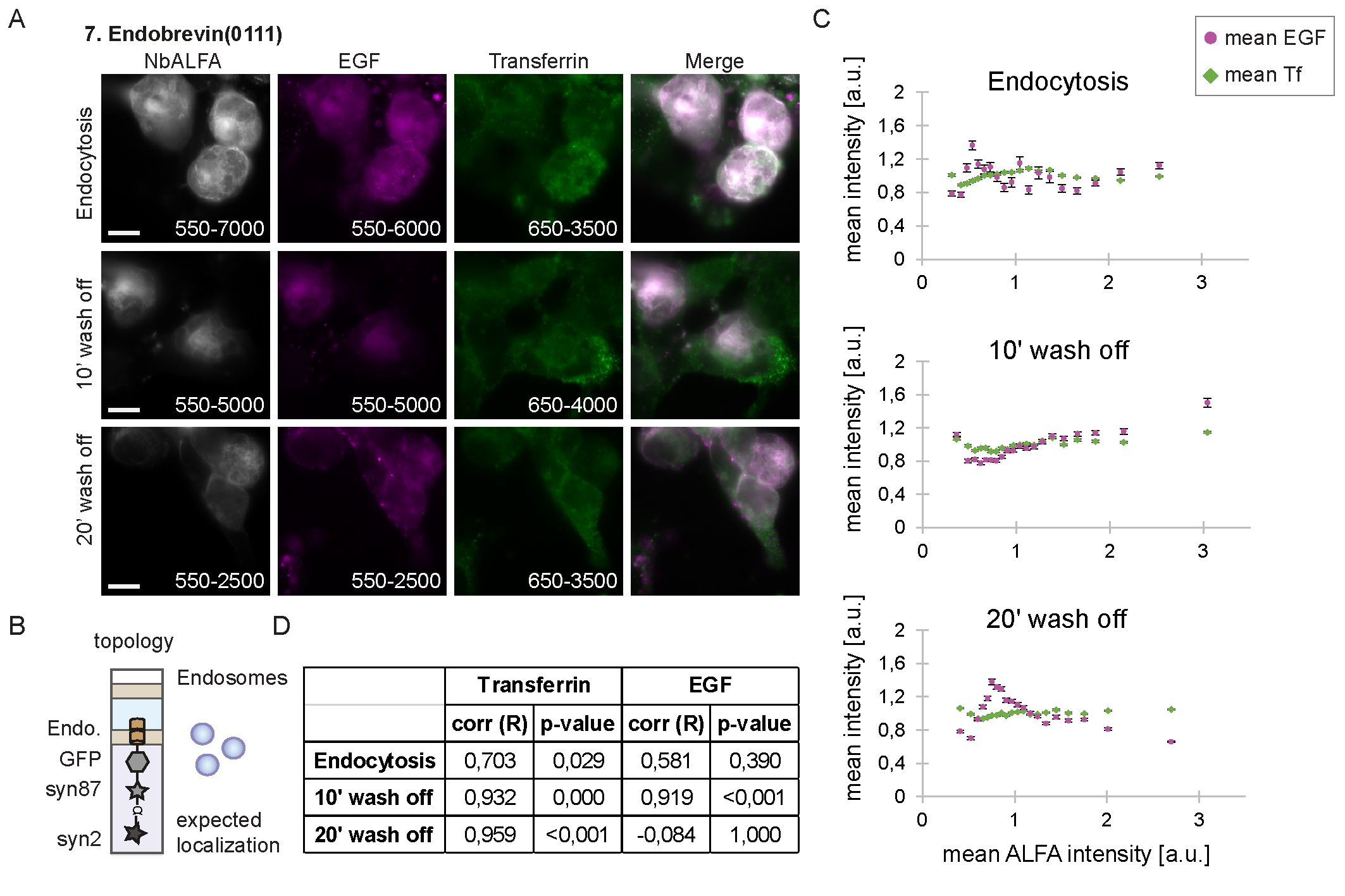

Supplement: S11 Fig — (A) Visualization of transferrin-Alexa488 (green) and EGF-Alexa647 (magenta), as well as the transfected protein, visualized with the ALFA nanobody (NbALFA) conjugated to AZdye568 (white). The 3 rows show the 10-minute pulse with the ligands (endocytosis), followed by the 10- and 20-minute chase (wash-off). To enable optimal visualization, the images are scaled differently, with the image scaling indicated in all panels. Scale bar: 20 μm. (B) The nanobarcoding scheme and the expected localization of the protein. (C) The NbALFA fluorescence intensity is plotted against the transferrin (green) and EGF (magenta) intensity, for all signals measured in 2 independent experiments, for all conditions. All intensities were normalized to the medians of the distributions and were then grouped in 20 bins of ALFA intensity, each containing similar numbers of values. The mean and SEM of each bin in the respective channels are plotted. The data underlying this Figure can be found in the S1 Data file, Sheet “SFig 11C_Endo,” available from http://dx.doi.org/10.17169/refubium-40101. (D) The Pearson’s correlation coefficients for the distributions from panel C are shown, with the p-values corrected for multiple testing using a Bonferroni correction. (PNG) [file pbio.3002427.s011.png]

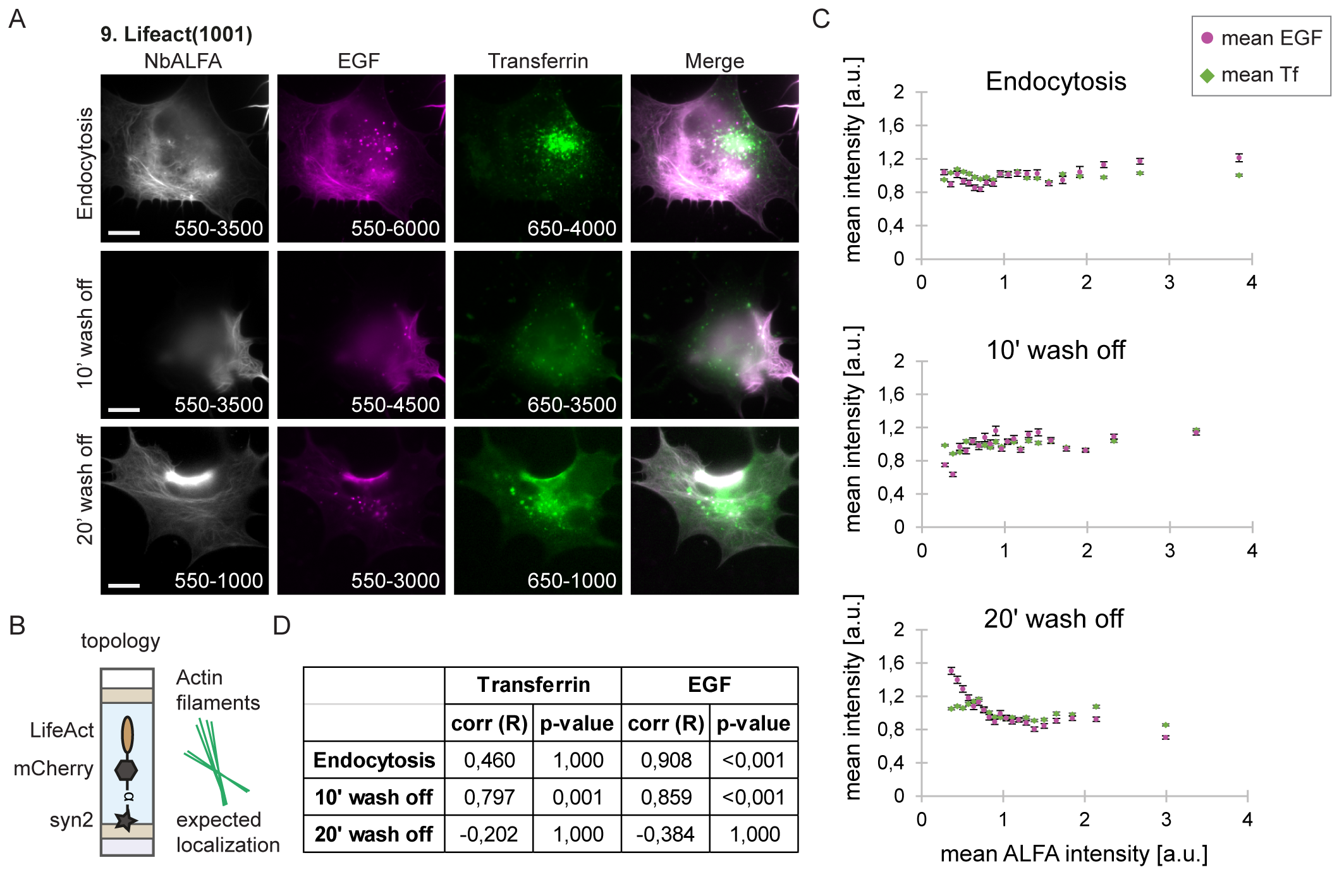

Supplement: S12 Fig — (A) Visualization of transferrin-Alexa488 (green) and EGF-Alexa647 (magenta), as well as the transfected protein, visualized with the ALFA nanobody (NbALFA) conjugated to AZdye568 (white). The 3 rows show the 10-minute pulse with the ligands (endocytosis), followed by the 10- and 20-minute chase (wash-off). To enable optimal visualization, the images are scaled differently, with the image scaling indicated in all panels. Scale bar: 20 μm. (B) The nanobarcoding scheme and the expected localization of the protein. (C) The NbALFA fluorescence intensity is plotted against the transferrin (green) and EGF (magenta) intensity, for all signals measured in 2 independent experiments, for all conditions. All intensities were normalized to the medians of the distributions and were then grouped in 20 bins of ALFA intensity, each containing similar numbers of values. The mean and SEM of each bin in the respective channels are plotted. The data underlying this Figure can be found in the S1 Data file, Sheet “SFig 12C_LifeAct,” available from http://dx.doi.org/10.17169/refubium-40101. (D) The Pearson’s correlation coefficients for the distributions from panel C are shown, with the p-values corrected for multiple testing using a Bonferroni correction. (TIF) [file pbio.3002427.s012.tif]

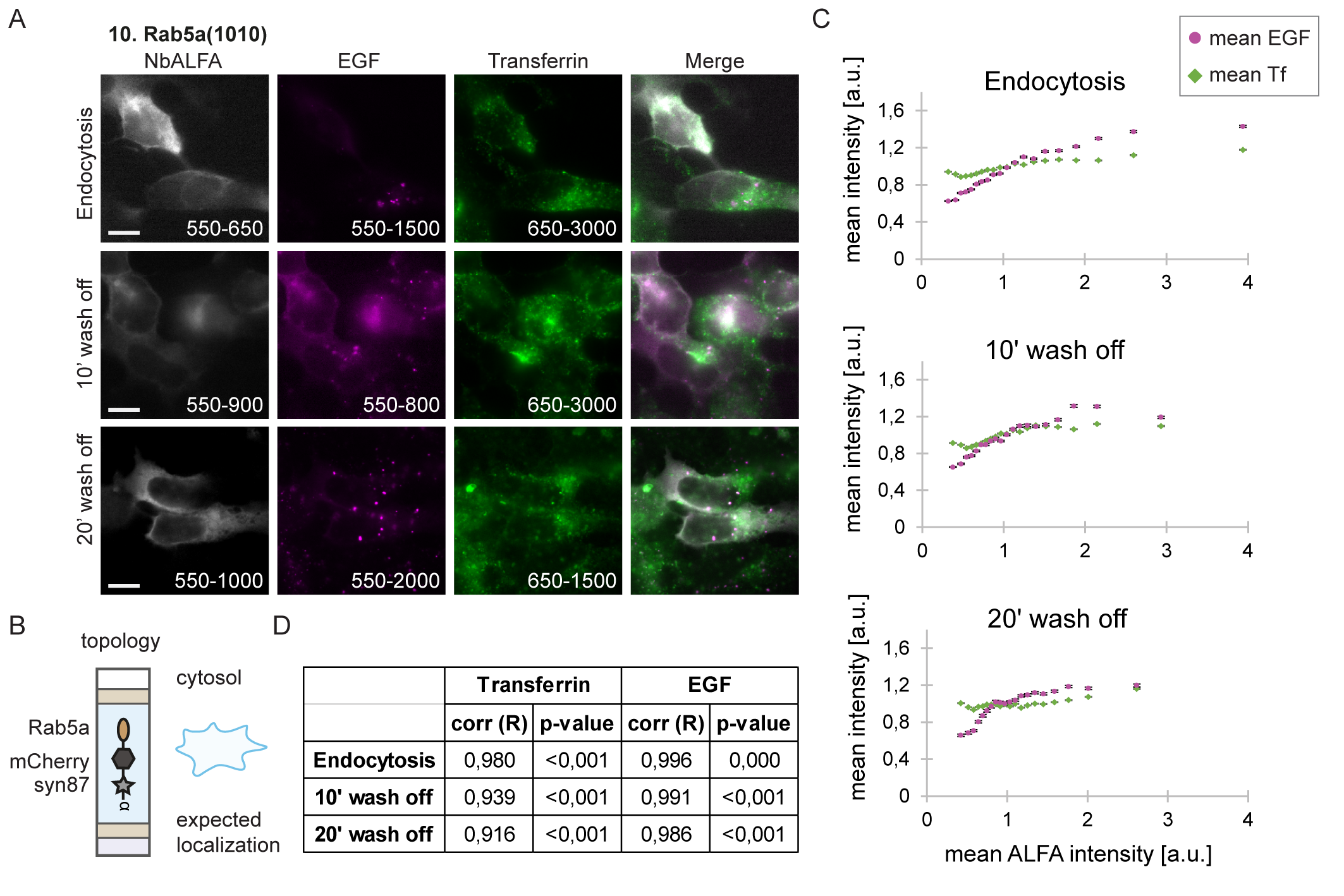

Supplement: S13 Fig — (A) Visualization of transferrin-Alexa488 (green) and EGF-Alexa647 (magenta), as well as the transfected protein, visualized with the ALFA nanobody (NbALFA) conjugated to AZdye568 (white). The 3 rows show the 10-minute pulse with the ligands (endocytosis), followed by the 10- and 20-minute chase (wash-off). To enable optimal visualization, the images are scaled differently, with the image scaling indicated in all panels. Scale bar: 20 μm. (B) The nanobarcoding scheme and the expected localization of the protein. (C) The NbALFA fluorescence intensity is plotted against the transferrin (green) and EGF (magenta) intensity, for all signals measured in 2 independent experiments, for all conditions. All intensities were normalized to the medians of the distributions and were then grouped in 20 bins of ALFA intensity, each containing similar numbers of values. The mean and SEM of each bin in the respective channels are plotted. The data underlying this Figure can be found in the S1 Data file, Sheet “SFig 13C_Rab5a,” available from http://dx.doi.org/10.17169/refubium-40101. (D) The Pearson’s correlation coefficients for the distributions from panel C are shown, with the p-values corrected for multiple testing using a Bonferroni correction. (TIF) [file pbio.3002427.s013.tif]

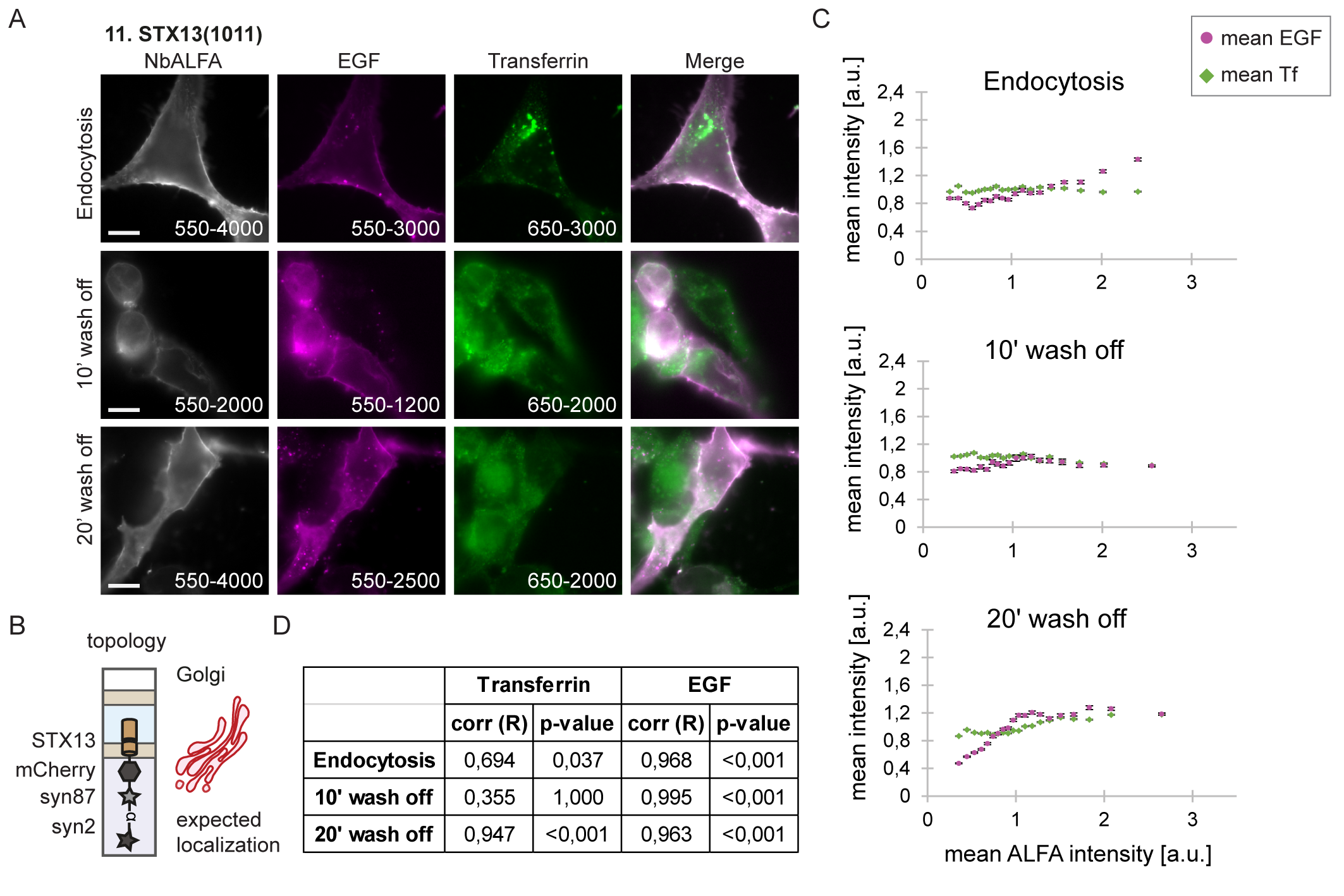

Supplement: S14 Fig — (A) Visualization of transferrin-Alexa488 (green) and EGF-Alexa647 (magenta), as well as the transfected protein, visualized with the ALFA nanobody (NbALFA) conjugated to AZdye568 (white). The 3 rows show the 10-minute pulse with the ligands (endocytosis), followed by the 10- and 20-minute chase (wash-off). To enable optimal visualization, the images are scaled differently, with the image scaling indicated in all panels. Scale bar: 20 μm. (B) The nanobarcoding scheme and the expected localization of the protein. (C) The NbALFA fluorescence intensity is plotted against the transferrin (green) and EGF (magenta) intensity, for all signals measured in 2 independent experiments, for all conditions. All intensities were normalized to the medians of the distributions and were then grouped in 20 bins of ALFA intensity, each containing similar numbers of values. The mean and SEM of each bin in the respective channels are plotted. The data underlying this Figure can be found in the S1 Data file, Sheet “SFig 14C_STX13,” available from http://dx.doi.org/10.17169/refubium-40101. (D) The Pearson’s correlation coefficients for the distributions from panel C are shown, with the p-values corrected for multiple testing using a Bonferroni correction. (TIF) [file pbio.3002427.s014.tif]

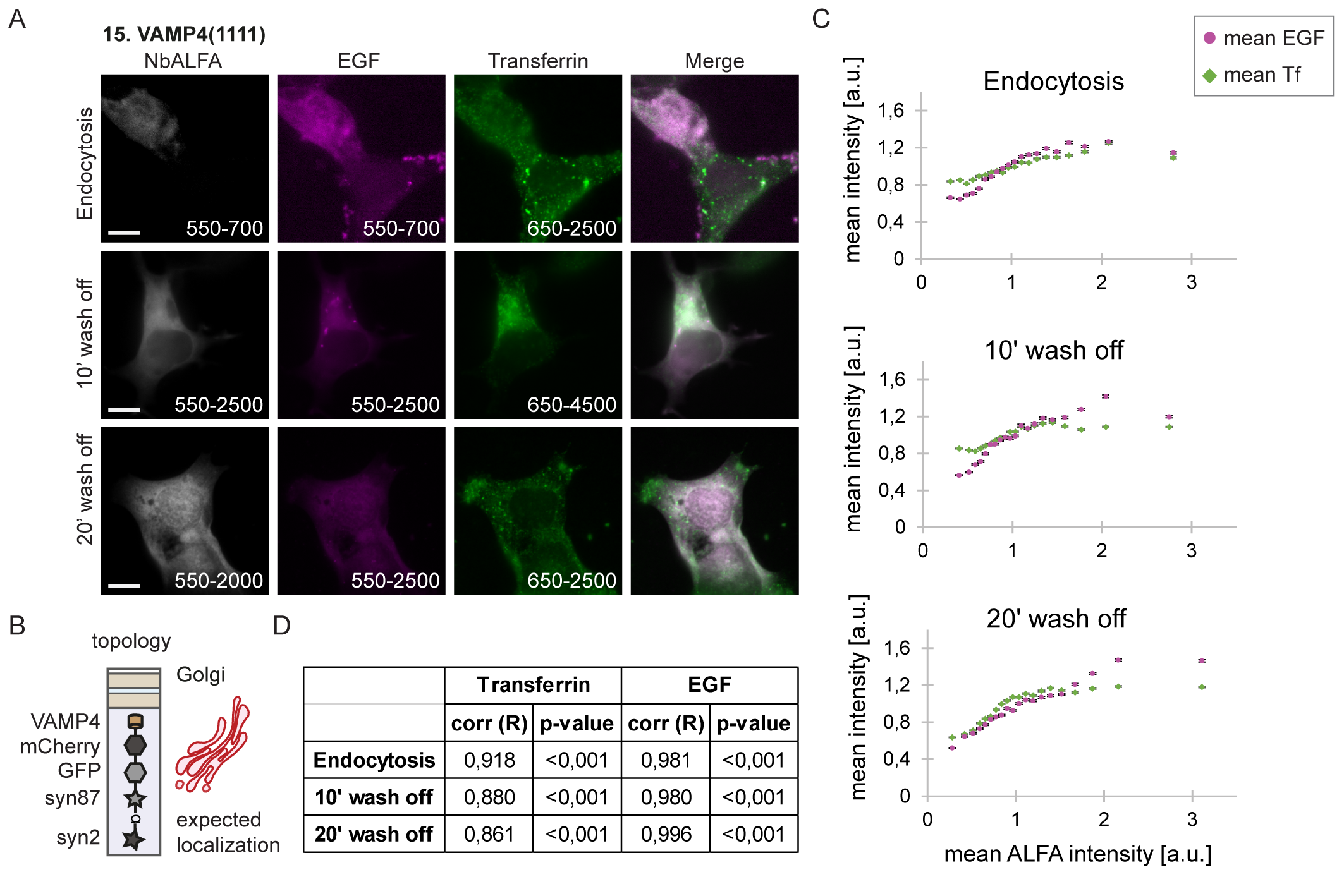

Supplement: S15 Fig — (A) Visualization of transferrin-Alexa488 (green) and EGF-Alexa647 (magenta), as well as the transfected protein, visualized with the ALFA nanobody (NbALFA) conjugated to AZdye568 (white). The 3 rows show the 10-minute pulse with the ligands (endocytosis), followed by the 10- and 20-minute chase (wash-off). To enable optimal visualization, the images are scaled differently, with the image scaling indicated in all panels. Scale bar: 20 μm. (B) The nanobarcoding scheme and the expected localization of the protein. (C) The NbALFA fluorescence intensity is plotted against the transferrin (green) and EGF (magenta) intensity, for all signals measured in 2 independent experiments, for all conditions. All intensities were normalized to the medians of the distributions and were then grouped in 20 bins of ALFA intensity, each containing similar numbers of values. The mean and SEM of each bin in the respective channels are plotted. The data underlying this Figure can be found in the S1 Data file, Sheet “SFig 15C_VAMP4,” available from http://dx.doi.org/10.17169/refubium-40101. (D) The Pearson’s correlation coefficients for the distributions from panel C are shown, with the p-values corrected for multiple testing using a Bonferroni correction. (TIF) [file pbio.3002427.s015.tif]

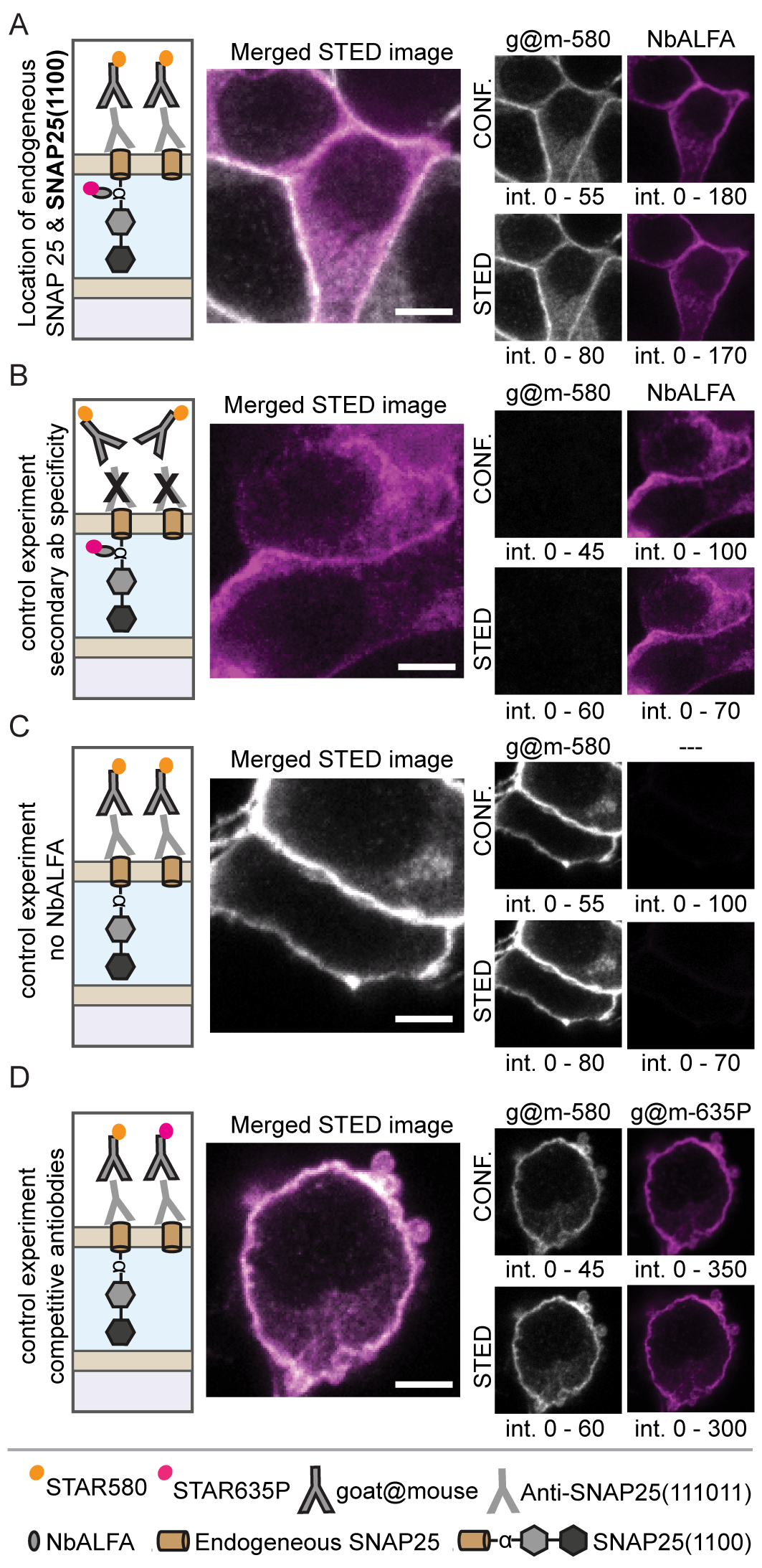

Supplement: S16 Fig — (A) Visualization of both endogenous SNAP25 and SNAP25(1100) using SNAP25 specific primary and secondary antibodies, plus NbALFA. (B, C) Negative control experiments, leaving out either primary antibodies (B) or NbALFA (C). (D) Imaging control, using a mixture of the same secondary antibody with 2 distinct fluorophores (targeting both endogenous SNAP25 and SNAP25(1100)), to provide a visual indication of the maximum expected colocalization. Bottom part of the figure: legend for used symbols and schemes. Scale bars: 2.5 μm. For quantification, see S17 Fig. (TIF) [file pbio.3002427.s016.tif]

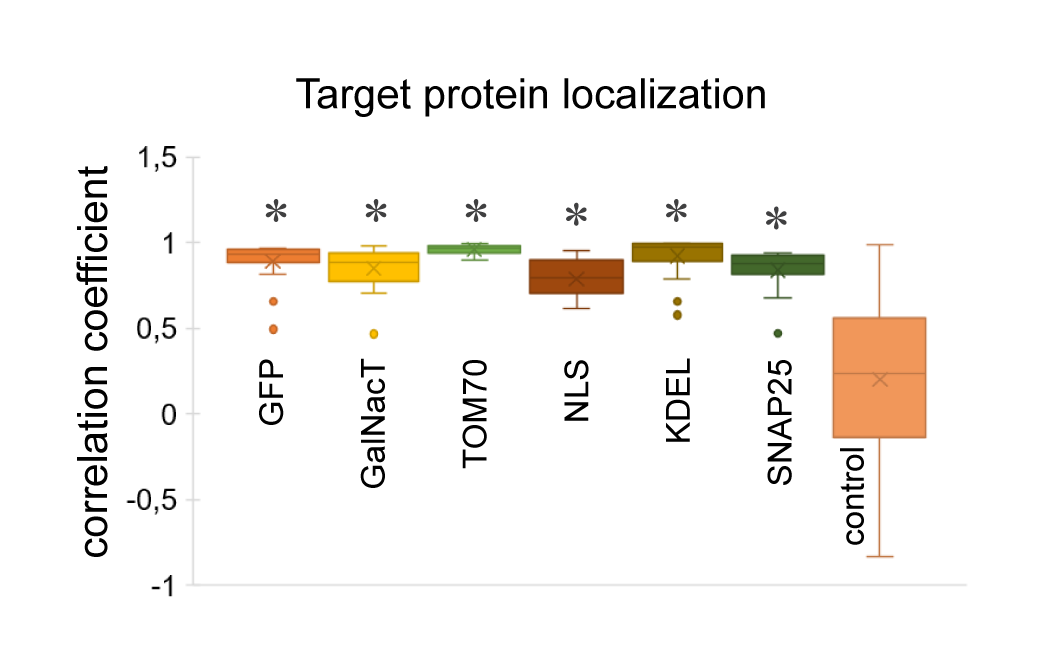

Supplement: S17 Fig — The images from S5 and S16 Figs were analyzed by measuring the Pearson’s correlation coefficient in different image regions. The box plot indicates the respective values, compared to a control, consisting of similar measurements across the same regions in the protein-of-interest channel, and mirrored regions in the compartment channel. All proteins show a colocalization that is significantly above the control values (Kruskal–Wallis test followed by Tukey post hoc test, p < 0.006 for all proteins). The data underlying this Figure can be found in the S1 Data file, Sheet “SFig 17_all_loc_func,” available from http://dx.doi.org/10.17169/refubium-40101. (TIF) [file pbio.3002427.s017.tif]

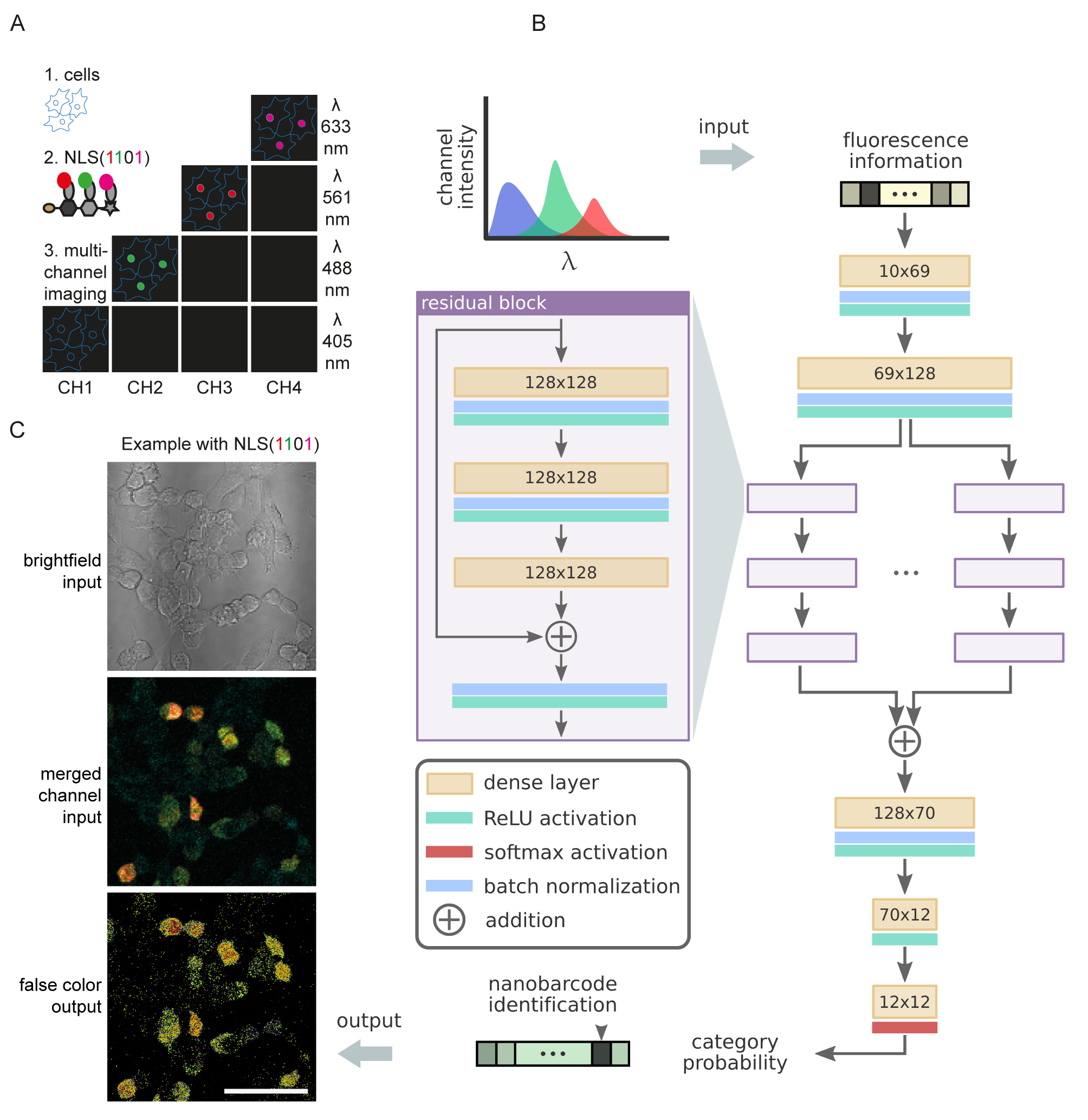

Supplement: S18 Fig — (A) Schematic representation of experimental protocol for obtaining multichannel images of HEK293 cells transfected with a single protein construct. HEK293 cells are seeded (1) and transfected with the necessary DNA plasmids. After an incubation of at least 14 hours, the HEK293 cells, now expressing the protein constructs, are fixed and stained with nanobodies (2). Multichannel images from the respective cells (3) are used for the training of a neuronal network. Wavelengths of excitation lasers used: λ = 405 nm, λ = 488 nm, λ = 561 nm, and λ = 633 nm. Emission channels used: 417–485 nm (CH1), 495–553 nm (CH2), 573–631 nm (CH3), and 641–729 nm (CH4). (B) Architecture of the deep network used for protein identification from channel intensity values pertaining to each pixel. For the dense layers, given numbers indicate input and output dimensions. The network contains 4 parallel branches in the middle (2 are shown), the outputs of which are summed and processed by the final layers. The branches are composed of sequential residual blocks with skip connections bypassing triplets of layers, as shown in the expansion panel to the left (further details in Methods section “Deep neural network-based protein identification”). (C) The output probability distributions of the network are used to render false color images that contain information on the identified proteins in each pixel. Scale bars: 50 μm. (TIF) [file pbio.3002427.s018.tif]

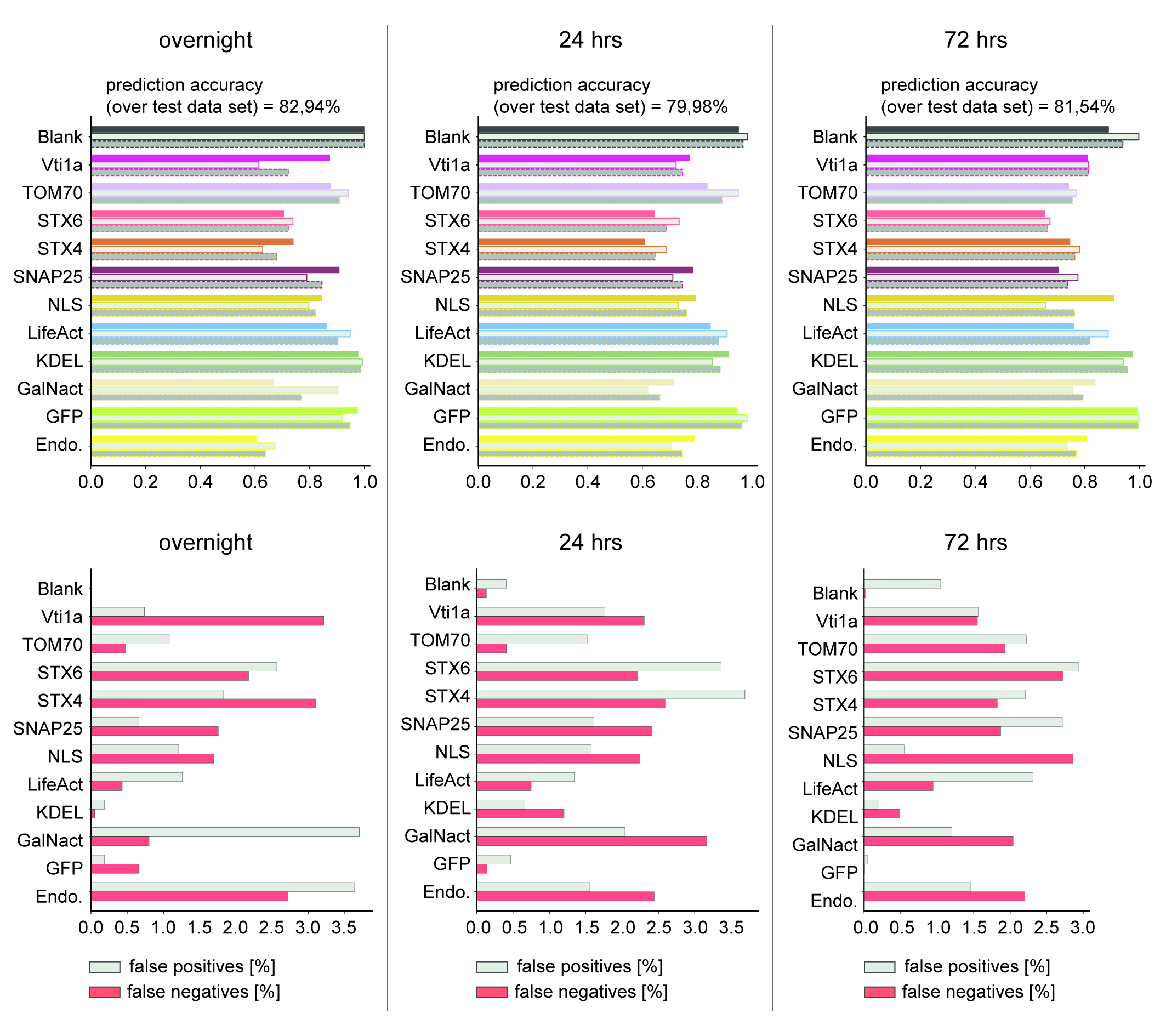

Supplement: S19 Fig — Data are shown for overnight, 24 hours and 72 hours protein expression, respectively. The data underlying this Figure are available as file “FigS19.xlsx” from http://dx.doi.org/10.17169/refubium-40101. The metrics for 48 hours are shown in Fig 2C and 2D. (TIF) [file pbio.3002427.s019.tif]

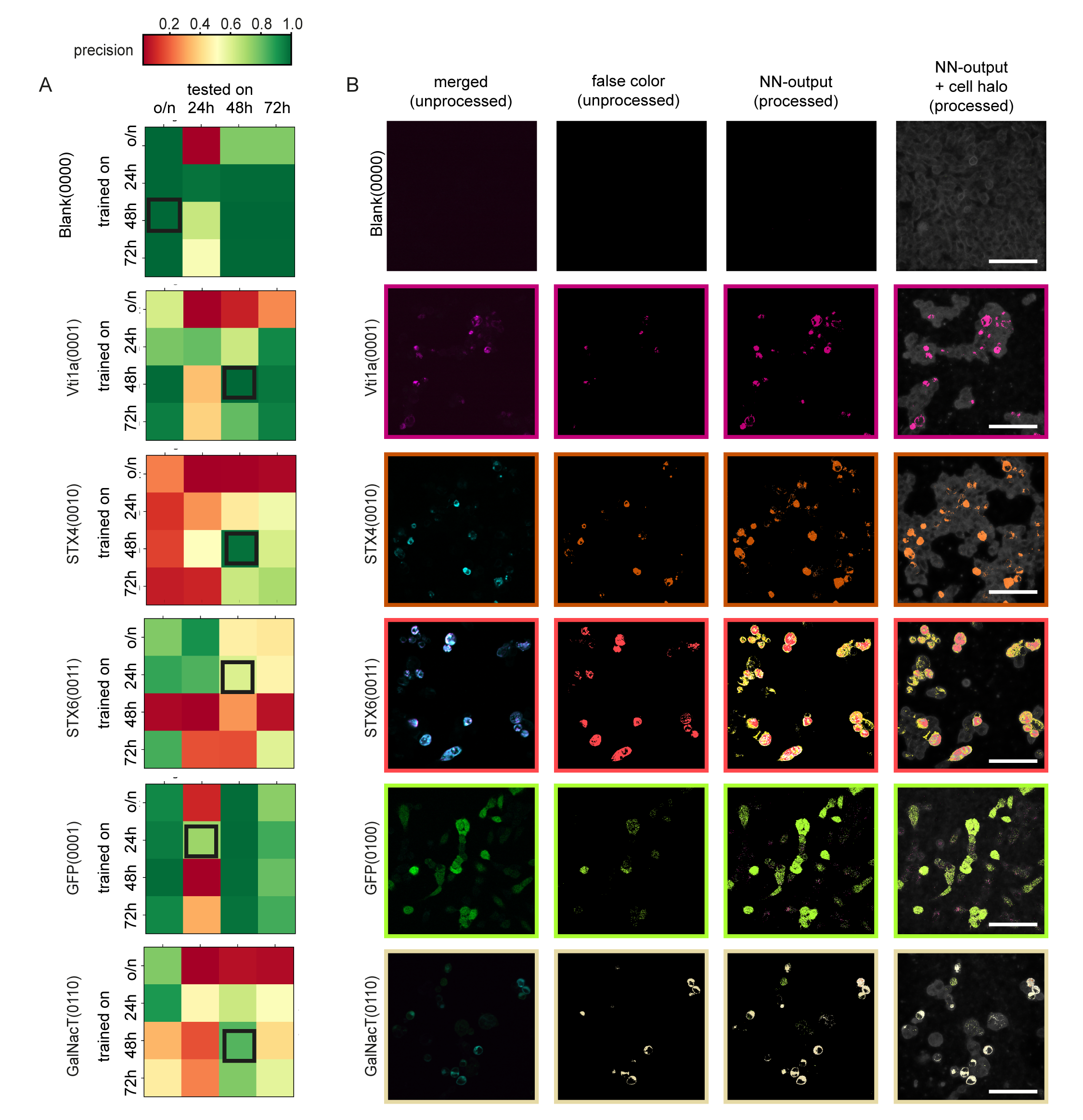

Supplement: S20 Fig — (A) The prediction accuracy matrix of trained deep networks, estimated over all the images in the dataset. To increase the complexity of the training and testing procedure, we expressed each construct for different time periods, and we then trained and tested the deep networks with all of these different datasets. Each row corresponds to a separate network that has been trained solely on the given dataset. Columns are the average pixel-wise prediction accuracy, assuming that all the pixels picked by the network in an image should belong to the protein with which the cells have been transfected. The given accuracy values may include effects of misexpressed proteins, weak fluorescence signals, and imaging noise. (B) From left to right, first column: merged channels (405 nm/CH1, 488 nm/CH2, 561 nm/CH3, 633 nm/CH4), before being processed by the network. Second column: images produced by assigning false colors to bright pixels, assuming that all the proteins in the image exactly match the given nanobarcode. Third column: output of the deep network, with each pixel given the false color representing the protein picked by the network. Colors are scaled based on class probabilities (Fig 2). Fourth column: false color output of the network overlaid on the gray “cell halos” produced from the brightfield images. Brightfield images have been processed to remove noise and background gradients and to enhance the contrast. (C, D) As (A) and (B), for additional nanobarcode proteins. The data underlying this Figure are available as file “FigS20_AC.xlsx” from http://dx.doi.org/10.17169/refubium-40101. (TIFF) [file pbio.3002427.s020.tiff]

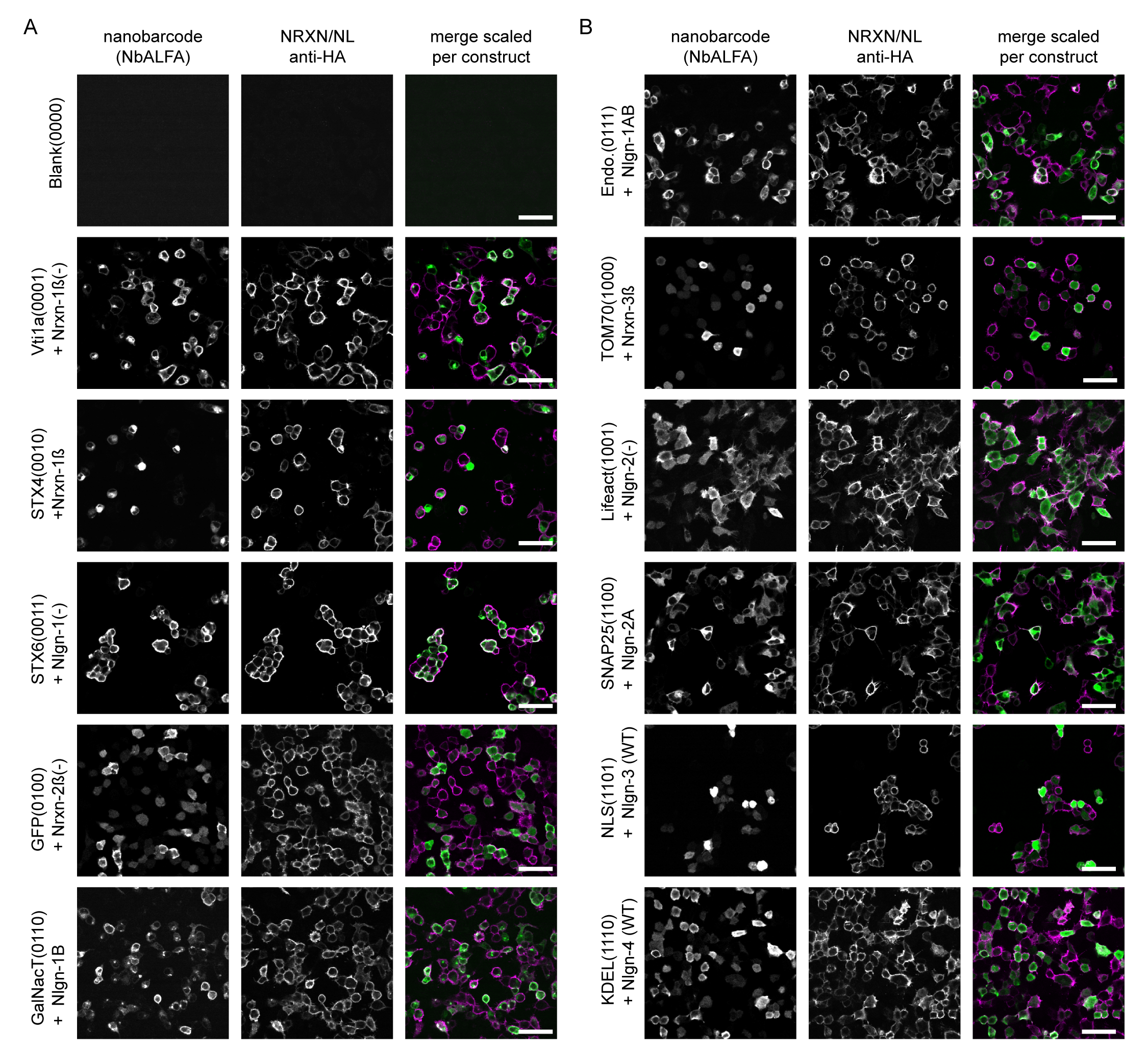

Supplement: S21 Fig — To enable an analysis of Nrxn and Nlgn pairing, we coexpressed different Nrxn and Nlgn constructs with specific nanobarcode proteins. This enables us to provide the different Nrxn- and Nlgn-containing cells with a recognizable identity, without having to modify the additional proteins themselves by nanobarcode tagging. However, this implies that we need to verify whether the majority of Nrxn- or Nlgn-expressing cells also express the respective nanobarcode proteins. (A, B) Nanobody staining with anti-ALFA-Atto488 reveals cells expressing protein constructs with nanobarcodes. Antibody staining with mouse-anti-HA and Cy3-anti-mouse reveals cells expressing NRXN or NL constructs with HA-tags. An overlay of both signals (anti-ALFA in green and anti-HA in magenta) indicates double-transfected cells, which make up the majority of all cells. N = 2 independent experiments for each protein combination. Scale bars: 50 μm. (TIF) [file pbio.3002427.s021.tif]
