# Supplementary material for: Protein nanobarcodes enable single-step multiplexed fluorescence imaging
Source: PLoS Biol. 2023 Dec 11;21(12):e3002427. doi: 10.1371/journal.pbio.3002427 (PMC10735187; doi:10.1371/journal.pbio.3002427)
Supplement: S1 Table — (DOCX) [file pbio.3002427.s022.docx]

| **Protein** | **Validation in literature (see reference list, below)** | **Validation in this manuscript** |
| --- | --- | --- |
| 1. Vti1a(0001) | - [1–3] | EGF and Transferrin experiments  (Suppl. Fig. 7) |
| 1. Syntaxin4(0010) | - [4,5] - Synuclein Nanobody epitopes / tags are short [6–8] | EGF and Transferrin experiments  (Suppl. Fig. 8) |
| 1. Syntaxin6(0011) | - [1] - Synuclein Nanobody epitopes / tags are short [6–8] | EGF and Transferrin experiments (Suppl. Fig. 9) |
| 1. GFP(0100) | - No known function, therefore no functional validation needed; only verification of localization needed. | Colocalization assays  (Suppl. Fig. 5) |
| 1. STX7(0101) | - [9–11] and probably others as well | EGF and Transferrin experiments  (Suppl. Fig. 10) |
| 1. GalNacT(0110) | - Localization is related to fuction, so was investigated here [12]. | Colocalization assays  (Suppl. Fig. 5) |
| 1. Endobrevin(0111) | - [13,14] | EGF and Transferrin experiments  (Suppl. Fig. 11) |
| 1. TOM70(1000) | - No known function, therefore no functional validation needed; only verification of localization needed. | Colocalization assays  (Suppl. Fig. 5) |
| 1. LifeAct(1001) | - Actin-binding molecule, designed to be tagged [15]. | EGF and Transferrin experiments  (Suppl. Fig. 12) |
| 1. Rab5a(1010) | - [1,2,16,17] | EGF and Transferrin experiments  (Suppl. Fig. 13) |
| 1. STX13(1011) |  | EGF and Transferrin experiments  (Suppl. Fig. 14) |
| 1. SNAP25(1100) | - Tested in cell lines [1,18–20] | STED SNAP25 (PC 12 cells)  (Suppl. Fig. 16) |
| 1. NLS(1101) | - No known function, therefore no functional validation needed; only verification of localization needed. | Colocalization assays  (Suppl. Fig. 5) |
| 1. KDEL(1110) | - No known function, therefore no functional validation needed; only verification of localization needed. | Colocalization assays  (Suppl. Fig. 5) |
| 1. VAMP4(1111) | - [1,21,22] | EGF and Transferrin experiments  (Suppl. Fig. 15) |

**Supplementary Table 1. Protein tag validation in literature and/or in this manuscript.**

**Reference List for Supplementary Table 1.**

1. Reshetniak S, Ußling J, Perego E, Rammner B, Schikorski T, Fornasiero EF, et al. A comparative analysis of the mobility of 45 proteins in the synaptic bouton. EMBO J. 2020;39. doi:10.15252/EMBJ.2020104596

2. Hoopmann P, Punge A, Barysch S V., Westphal V, Bückers J, Opazo F, et al. Endosomal sorting of readily releasable synaptic vesicles. Proc Natl Acad Sci U S A. 2010;107: 19055–19060. doi:10.1073/PNAS.1007037107/-/DCSUPPLEMENTAL/SM01.MOV

3. Ramirez DMO, Khvotchev M, Trauterman B, Kavalali ET. Vti1a identifies a vesicle pool that preferentially recycles at rest and maintains spontaneous neurotransmission. Neuron. 2012;73: 121–134. doi:10.1016/J.NEURON.2011.10.034

4. Kalwat MA, Wiseman DA, Luo W, Wang Z, Thurmond DC. Gelsolin Associates with the N Terminus of Syntaxin 4 to Regulate Insulin Granule Exocytosis. Mol Endocrinol. 2012;26: 128–141. doi:10.1210/ME.2011-1112

5. Takuma T, Arakawa T, Okayama M, Mizoguchi I, Tanimura A, Tajima Y. Trafficking of Green Fluorescent Protein-Tagged SNARE Proteins in HSY Cells. J Biochem. 2002;132: 729–735. doi:10.1093/OXFORDJOURNALS.JBCHEM.A003280

6. De Genst EJ, Guilliams T, Wellens J, Day EM, Waudby CA, Meehan S, et al. Structure and properties of a complex of α-synuclein and a single-domain camelid antibody. J Mol Biol. 2010;402: 326–343. doi:10.1016/J.JMB.2010.07.001

7. El-Turk F, Newby FN, De Genst E, Guilliams T, Sprules T, Mittermaier A, et al. Structural Effects of Two Camelid Nanobodies Directed to Distinct C-Terminal Epitopes on α-Synuclein. Biochemistry. 2016;55: 3116–3122. doi:10.1021/ACS.BIOCHEM.6B00149

8. Guilliams T, El-Turk F, Buell AK, O’Day EM, Aprile FA, Esbjörner EK, et al. Nanobodies Raised against Monomeric α-Synuclein Distinguish between Fibrils at Different Maturation Stages. J Mol Biol. 2013;425: 2397–2411. doi:10.1016/J.JMB.2013.01.040

9. Prekeris R, Yang B, Oorschot V, Klumperman J, Scheller RH. Differential roles of syntaxin 7 and syntaxin 8 in endosomal trafficking. Mol Biol Cell. 1999;10: 3891–3908. doi:10.1091/MBC.10.11.3891

10. Mashima H, Suzuki J, Hirayama T, Yoshikumi Y, Ohno H, Ohnishi H, et al. Involvement of vesicle-associated membrane protein 7 in human gastric epithelial cell vacuolation induced by Helicobacter pylori-produced VacA. Infect Immun. 2008;76: 2296–2303. doi:10.1128/IAI.01573-07

11. Mori Y, Takenaka K ichiro, Fukazawa Y, Takamori S. The endosomal Q-SNARE, Syntaxin 7, defines a rapidly replenishing synaptic vesicle recycling pool in hippocampal neurons. Commun Biol 2021 41. 2021;4: 1–13. doi:10.1038/s42003-021-02512-4

12. Becker JL, Tran DT, Tabak LA. Members of the GalNAc-T family of enzymes utilize distinct Golgi localization mechanisms. Glycobiology. 2018;28: 841. doi:10.1093/GLYCOB/CWY071

13. Nagamatsu S, Nakamichi Y, Watanabe T, Matsushima S, Yamaguchi S, Ni J, et al. Localization of cellubrevin-related peptide, endobrevin, in the early endosome in pancreatic β cells and its physiological function in exo-endocytosis of secretory granules. J Cell Sci. 2001;114: 219–227. doi:10.1242/JCS.114.1.219

14. Okayama M, Arakawa T, Tanimura A, Mizoguchi I, Tajima Y, Takuma T. Role of VAMP8/endobrevin in Constitutive Exocytotic Pathway in HeLa Cells. Cell Struct Funct. 2009;34: 115–125. doi:10.1247/CSF.09013

15. Riedl J, Crevenna AH, Kessenbrock K, Yu JH, Neukirchen D, Bista M, et al. Lifeact: a versatile marker to visualize F-actin. Nat Methods. 2008;5: 605–607. doi:10.1038/NMETH.1220

16. Bethani I, Lang T, Geumann U, Sieber JJ, Jahn R, Rizzoli SO. The specificity of SNARE pairing in biological membranes is mediated by both proof-reading and spatial segregation. EMBO J. 2007;26: 3981–3992. doi:10.1038/SJ.EMBOJ.7601820

17. Pavlos NJ, Grønborg M, Riedel D, Chua JJE, Boyken J, Kloepper TH, et al. Quantitative analysis of synaptic vesicle Rabs uncovers distinct yet overlapping roles for Rab3a and Rab27b in Ca2+-triggered exocytosis. J Neurosci. 2010;30: 13441–13453. doi:10.1523/JNEUROSCI.0907-10.2010

18. An SJ, Almers W. Tracking SNARE complex formation in live endocrine cells. Science. 2004;306: 1042–1046. doi:10.1126/SCIENCE.1102559

19. Halemani ND, Bethani I, Rizzoli SO, Lang T. Structure and dynamics of a two-helix SNARE complex in live cells. Traffic. 2010;11: 394–404. doi:10.1111/J.1600-0854.2009.01020.X

20. Rickman C, Medine CN, Dun AR, Moulton DJ, Mandula O, Halemani ND, et al. t-SNARE protein conformations patterned by the lipid microenvironment. J Biol Chem. 2010;285: 13535–13541. doi:10.1074/JBC.M109.091058

21. Nicholson-Fish JC, Kokotos AC, Gillingwater TH, Smillie KJ, Cousin MA. VAMP4 Is an Essential Cargo Molecule for Activity-Dependent Bulk Endocytosis. Neuron. 2015;88: 973–984. doi:10.1016/J.NEURON.2015.10.043

22. Tran THT, Zeng Q, Hong W. VAMP4 cycles from the cell surface to the trans-Golgi network via sorting and recycling endosomes. J Cell Sci. 2007;120: 1028–1041. doi:10.1242/jcs.03387
