# Supplementary material for: Protein nanobarcodes enable single-step multiplexed fluorescence imaging
Source: PLoS Biol. 2023 Dec 11;21(12):e3002427. doi: 10.1371/journal.pbio.3002427 (PMC10735187; doi:10.1371/journal.pbio.3002427)
Supplement: S2 Table — The first 19 antibodies listed are primary antibodies. Antibodies 20–23 are secondary antibodies. See Methods section for further information about the staining procedures. (DOCX) [file pbio.3002427.s023.docx]

|  | **Antibody** | **Target protein** | **Species** | **Dilution from stock** | **Company** | **Cat#N** |
| --- | --- | --- | --- | --- | --- | --- |
| 01 | Anti-Vti1a | Vti1a | Rabbit polyclonal | ICC 1:500 | Synaptic System | 165 002 |
| 02 | Anti-Syntaxin 4 | Syntaxin 4 | Rabbit polyclonal | ICC 1:300 | Synaptic Systems | 110 042 |
| 03 | Anti-Syntaxin 6 | Syntaxin 6 | Rabbit  polyclonal | ICC 1:500 | Synaptic Systems | 110 062 |
| 04 | GFP, does not have a target protein, see Supplementary Figure 3 for validation of epitope | | | | | |
| 05 | Anti-Syntaxin 7 | Syntaxin 7 | Rabbit polyclonal | ICC 1:300 | Synaptic Systems | 110 073 |
| 06 | Anti-GM130 | GM130 | Rabbit polyclonal | ICC 1:300 | Sigma-  Aldrich | G7295 |
| 07 | Anti-Endobrevin | Endobrevin | Rabbit polyclonal | ICC 1:300 | Synaptic Systems | 104 303 |
| 08 | Anti-TOMM20 | Sigma | Mouse monoclonal | ICC 1:200 | Sigma- Aldrich | WH0009804M1 |
| 09 | Anti-Beta-Actin | Beta-Actin | Mouse polyclonal | ICC 1:75 | Sigma- Aldrich | A1978 |
| 10 | Anti-Rab5a | Rab5a | Rabbit monoclonal | ICC 1:250 | Abcam | ab199530 |
| 11 | Anti-Syntaxin 12/13 | Syntaxin 12/13 | Rabbit polyclonal | ICC 1:300 | Synaptic System | 110 133 |
| 12 | Anti-SNAP 25 | SNAP 25 | Rabbit polyclonal | ICC 1:500 | Synaptic Systems | 111 002 |
| 13 | Anti-NFkappaB p65 | NLS | Rabbit monoclonal | ICC 1:200 | Biomol / Rockland | 600-401-271 |
| 14 | Anti-KDEL | KDEL | Mouse monoclonal | ICC 1:500 | Enzo Life Sciences | ADI-SPA-827-F |
| 15 | Anti-VAMP 4 | VAMP 4 | Rabbit polyclonal | ICC 1:300 | Synaptic Systems | N136 002 |
| 16 | Anti-HA | HA-Tag | Goat | ICC 1:250 | Novus Biologicals | NB600-362 |
| 17 | Anti-SNAP25 | SNAP25 | Mouse | ICC 1:200 | Synaptic Systems | 111011 |
| 18 | Anti-Calnexin | Calnexin | Rabbit plyclonal | ICC 1:200 | Abcam | Ab22595 |
| 19 | Anti-GM130 | GM130 | Mouse monoclonal | ICC 1:200 | BD Biosciences | 610822 clone 35/GM130 |
|  | Anti-TOMM20 | TOMM20 | Mouse monoclonal | ICC 1:200 | Proteintech | 11802-1-AP |
| 20 | Anti-Rabbit  IgG Cy5 | Anti-Rabbit IgG | Donkey | ICC1:500 | Dianova | 711-175-152 |
| 21 | Anti-Mouse  IgG Cy5 | Anti-Mouse IgG | Donkey | ICC 1:500 | Dianova | SEC-183329 |
| 22 | Anti-Mouse  IgG Star580 | Anti-Mouse IgG | Goat | ICC 1:100 | Abberior | ST580-1001-500µg |
| 23 | Anti-Rabbit IgG Star580 | Anti-Rabbit IgG | Goat | ICC 1:100 | Abberior | ST580-1002-500µg |

**Supplementary Table 2. Information about antibodies used for target protein validation purposes.** The first nineteen antibodies listed are primary antibodies. Antibodies 20-23 are secondary antibodies. See Methods section for further information about the staining procedures.
