# Supplementary material for: Protein nanobarcodes enable single-step multiplexed fluorescence imaging
Source: PLoS Biol. 2023 Dec 11;21(12):e3002427. doi: 10.1371/journal.pbio.3002427 (PMC10735187; doi:10.1371/journal.pbio.3002427)
Supplement: S3 Table — Number of confocal images obtained for each protein (with the given nanobarcode) as well as number of pixels that have been sampled for the deep learning dataset. All images have the same dimensions of 512 × 512 pixels. With 72-hour samples, each image contains 5 slices in a z-stack. Network training, validation, and testing are done only based on the subsampled pixels (with the numbers given in the last column), while the precision matrices in S20 Fig are obtained on full-frame images. (DOCX) [file pbio.3002427.s024.docx]

|  | **Protein** | **Time window** | **Number of Images** | **Total number of sampled pixels** |
| --- | --- | --- | --- | --- |
| 01 | Blank (no protein) | N/A | 24 | 2400000 |
| 02 | Endobrevin (0111) | overnight | 10 | 10000 |
|  |  | 24h | 12 | 10000 |
|  |  | 48h | 10 | 10000 |
|  |  | 72h | 5 | 10000 |
| 03 | GalNacT (0110) | overnight | 9 | 3224 |
|  |  | 24h | 12 | 10000 |
|  |  | 48h | 10 | 2340 |
|  |  | 72h | 5 | 10000 |
| 04 | GFP (0100) | overnight | 10 | 10000 |
|  |  | 24h | 12 | 10000 |
|  |  | 48h | 5 | 10000 |
|  |  | 72h | 5 | 10000 |
| 05 | KDEL (1110) | overnight | 5 | 10000 |
|  |  | 24h | 12 | 10000 |
|  |  | 48h | 10 | 10000 |
|  |  | 72h | 5 | 10000 |
| 06 | Lifeact (1001) | overnight | 5 | 10000 |
|  |  | 24h | 12 | 10000 |
|  |  | 48h | 10 | 10000 |
|  |  | 72h | 5 | 10000 |
| 07 | NLS (1101) | overnight | 10 | 10000 |
|  |  | 24h | 12 | 10000 |
|  |  | 48h | 10 | 10000 |
|  |  | 72h | 5 | 10000 |
| 08 | SNAP25 (1100) | overnight | 5 | 5412 |
|  |  | 24h | 12 | 10000 |
|  |  | 48h | 10 | 10000 |
|  |  | 72h | 5 | 10000 |
| 09 | STX4 (0010) | overnight | 10 | 10000 |
|  |  | 24h | 12 | 10000 |
|  |  | 48h | 10 | 10000 |
|  |  | 72h | 5 | 10000 |
| 10 | STX6 (0011) | overnight | 10 | 10000 |
|  |  | 24h | 12 | 10000 |
|  |  | 48h | 11 | 10000 |
|  |  | 72h | 5 | 10000 |
| 11 | TOM70 (1000) | overnight | 9 | 10000 |
|  |  | 24h | 7 | 10000 |
|  |  | 48h | 10 | 10000 |
|  |  | 72h | 5 | 1000 |
| 12 | Vti1a (1000) | overnight | 9 | 1033 |
|  |  | 24h | 12 | 10000 |
|  |  | 48h | 10 | 10000 |
|  |  | 72h | 5 | 3825 |

**Supplementary Table 3. Summary of the image data used for training and testing the deep network.** Number of confocal images obtained for each protein (with the given nanobarcode) as well as number of pixels that have been sampled for the deep learning dataset. All images have the same dimensions of 512x512 pixels. With 72 h samples, each image contains 5 slices in a z-stack. Network training, validation, and testing are done only based on the sub-sampled pixels (with the numbers given in the last column), while the precision matrices in Supplementary Fig 20 are obtained on full-frame images.
