# Supplementary material for: Protein nanobarcodes enable single-step multiplexed fluorescence imaging
Source: PLoS Biol. 2023 Dec 11;21(12):e3002427. doi: 10.1371/journal.pbio.3002427 (PMC10735187; doi:10.1371/journal.pbio.3002427)
Supplement: Attachment — Submitted filename: Response_to_reviewers_revised_manuscript.pdf [file pbio.3002427.s027.pdf]

Replies to the Reviewer comments

Reviewer #1:

I have read the authors' responses and the revised manuscript with a lot of interest. I am particularly impressed with the authors' thorough implementation of functional assays to test the effect of their nanobarcodes on the different proteins they image. I would like to thank the reviewers for their extensive work on the revised manuscript - which must have been extensive - but, I can attest now that the revised manuscript is much better poised for publication and that their technology will have a profound impact in the field.

We thank the Reviewer for his/her comments.

Reviewer #3:

The authors have carried out an in-depth revision of the manuscript. They have addressed most of the reviewers' comments and have greatly improved the description of the analysis method and the accessibility of their code. The Github repository where the code is shared is well documented. The notebooks are easy to use and run smoothly when following the set-up described on Github. The code is well documented, commented, and clearly partitioned into functions. The functions and their parameters correspond to what is described in the manuscript and the methods section.

We thank the Reviewer for considering our code with careful scrutiny, and are glad that the Reviewer found it convenient to use.

Major comment :
The effect of the spatial resolution on the reliability of the nanobarcoding approach and deep learning based analysis should be better demonstrated and described in the result section. A very short paragraph describing the aspect of having only one nanobarcode per pixel was included in the discussion but I think that this should be described with more details already with the presented results. For example, in the paragraph describing the results about the Nrxn and Nlgn interactions, it is not clear to me how pairs are identified and what is considered as being an interaction.

The interactions measured here are indeed between Nrxn and Nlgn. However, these molecules are not fluorescently labeled and their binding to each other is not visualized. What we actually visualize fluorescently is the identity of the respective pairs of cells. These cells are labeled by different nanobarcoded proteins.

If I understand it correctly, only a single nanobarcode can be associated to one pixel, so that the spatial resolution of the microscope and the distance between interacting proteins will influence the precision of the analysis approach. Was this controlled for the Nrxn and Nlgn pairs? How would the reliability of the technique be influenced by interacting proteins that are closer that the resolution limit of the chosen microscope?

As explained above, it is the cells that are visualized and not the Nrxns and Nlgns. The cells are far above the diffraction limit of our microscope. Interacting proteins that are closer than the resolution of the microscope would lead to a mixed identity for the respective pixel. Therefore, subcellular investigations using nanobarcodes should aim for higher resolution, as we indicated in the paper (page 12, lines 287-292).

Notebooks
Both notebooks fail to load the network weights on a CPU machine. I suggest adding an 'if torch.cuda.is_available' statement when loading the weights. When False, the torch.load function should have the argument 'map_location=torch.device('cpu')'.

We thank the reviewer for bringing this issue to our attention. We modified the call to *torch.load* function to automatically map to the correct device. The fixed version of the notebook is already pushed to the github repository.

When using the environment as provided, I get the error message "Error displaying widget" when running the last cells of both notebooks. Make sure the environment includes the libraries for displaying the widgets, if the widgets are necessary.

This has been due to a known compatibility issue between the notebook widget *tqdm* and the recent Jupyter update. We rolled back to using the text-based progress-bar of *tqdm* which circumvents the issue. The fixed version of the code is already pushed to the github repository.

Minor comments:
In Fig. 2a, it would be more helpful to have a less simplified version of the designed neural network, highlighting the principal structural elements of the network.

We agree with the Reviewer and have now added more structural details of the network to Figure 2A. We have already provided all the details in Supplementary Figure 18.

Fig. 2b in the merged input column, the contrast is very low, making it very difficult to compare the signal with the NN output.

We enhanced the contrast of the merged input images and replaced the images from the left column in Fig. 2B.

Fig. 3b : please describe the red boxes in the legend and make sure that they are useful (it is not clear to me with this version of the figure why they have been added to the right panel).

Since Fig. 3B does not contain red boxes, we think the Reviewer is referring to Fig. 4B (previously Fig. 3). The boxes have been present in the previous version of the manuscript. For our revised manuscript we changed the dashed lines of the red boxes into solid lines, to make the boxes more apparent. We now added the following description to the legend of panel B: "(red boxes depict typical cell contacts)"

Fig. 3c : define the green and magenta colors in the legend

The green and magenta colors are already defined in the legend of Fig. 4C: "Nanobarcode-proteins are shown in green (anti-ALFA-Atto488). Nrxn or Nlgn isoforms are shown in magenta (anti-HA & anti-goat-Cy3)."

Lines 99-100 The approach appeared to function well, as illustrated by the images in Fig 1F, in which the nanobarcodes can be easily differentiated by the human observer. I suggest rephrasing : As illustrated in Fig 1F, the nanobarcodes can be easily differentiated by the human observer.

We now changed the sentence according to the Reviewer's suggestion.

At line 167, authors should state why a trainable contrast modifier was needed and add a reference to support the use of such an approach in this context. Similarly, it is not clear on line 177 why 50 iterations of self-supervised contrast adaptation is required.

The trainable contrast-modifier is indeed an optional refinement step, and the user can control its invocation via the *n_optim_iter* argument in the *predict_from_image_file* function (if set to zero, the contrast-modifier will not be used at all).

We have thus edited the text to clarify the optional nature of contrast-modifier, how it actually helps at inference time, and why we used only 50 steps of training (page 8, lines 177-185).

Lines 194-195, please add references for the listed methods.

We provided the citations for all the listed methods.

Lines 198-203 : Performing the ablation study was very helpful to investigate the class-dependent performance of the network. However, I would suggest rephrasing the paragraph between lines 198-203 as reducing the number of classes in an ablation study has also an impact on the task complexity, which can positively impact the overall performance.

We revised the text and explained that the ablation generally increases the performance, but this increase is not uniform, and thus highlights the sensitivity of the prediction to different target classes (page 9, lines 217-221).

Line 206 : please define what is satisfactory precision. Was there a minimal precision that was identified in order for this method to be useful in the described experimental context?

We added text to explain our take on the satisfactory precision. For that, we provided statistics on the prediction precision on whole images (page 10, lines 226-233).

The added paragraph between lines 206 and 234 is difficult to follow as links between the sentences and with the main subject of the paper are often missing or unclear. I suggest reworking on this section of the paper to improve clarity.

We simplified the respective paragraph, while also explaining more clearly some notions that may only be well-known to synapse biologists (page 10, lines 236-242).

Line 1074 : cite the publication you are referring to in the statement The Nrxn/Nlgn codes, such as SS#4(+) refer to the respective splicing sites of the proteins, according to the literature.

We cited following publication as an example:
Südhof TC. Towards an Understanding of Synapse Formation. Neuron. 2018;100: 276–293. doi:10.1016/J.NEURON.2018.09.040

I suggest rewording the following sentences to improve clarity and readability:

Line 137 : The enhancements can only be limited, since the membrane trafficking pathways remain limited by the abundance of many other proteins, which are not overexpressed. We obtained this result for all proteins. Suppl. Fig 6 presents an overall view of the results, indicating the transferrin and EGF dynamics in all experiments, combined.

We have reworded this phrase, as follows: “The changes induced by the expression of barcoding proteins can only reach a moderate level, since the respective trafficking pathways remain limited by the abundance of other partner proteins, which are not overexpressed.”

Line 1020 (caption of figure 1) : Barcode epitopes recognized by fluorescent nanobodies are referred to as nanobarcodes and shown as "ones" in pseudo-colors that correspond to the fluorophores used. I suggest removing the added "are referred to as nanobarcodes"; from my understanding, the barcode epitopes (which are shown as "ones") are not referred to as nanobarcodes, it is the combination of four epitopes ("zeroes" and "ones") that are referred to as nanobarcodes.

We removed “are referred to as nanobarcodes” from the caption of Fig. 1.

Typos :
Line 54 : data sets -> datasets
Line 132 : (whose binding to actin should lead to a small, but measurable enhancement of actin dynamics, [17], Remove the comma and replace it with a parenthesis.
Line 146 : (just as lowering SNAP25 levels in heterozygous SNAP25+/- mice leads to very minor phenotypes, [19]. Remove the comma and replace it with a parenthesis.
Line 164 : Labelled -> labeled
Line 168 : and is trained self-supervised -> and is trained in a self-supervised manner
Line 176 : utilizing the same hardware, the inference, takes about 15 seconds -> utilizing the same hardware, the inference takes about 15 seconds (remove the extra comma)
Line 219 : (e.g. [22] but has only recently been related to the synapse [23]. Close the parenthesis.
Line 460 : A batch size of 458 is uses. Change uses to used.

We corrected those typos according to the Reviewer’s suggestion.
