# Supplementary material for: Protein nanobarcodes enable single-step multiplexed fluorescence imaging
Source: PLoS Biol. 2023 Dec 11;21(12):e3002427. doi: 10.1371/journal.pbio.3002427 (PMC10735187; doi:10.1371/journal.pbio.3002427)
Supplement: Attachment — Submitted filename: Response_to_reviewers.docx [file pbio.3002427.s026.docx]

**Replies to the Reviewer comments**

***Reviewer #1:*** *Jong-Bolm et al present a clever approach to multi-protein imaging via protein barcoding, potentially enabling the detection of more than 10 different proteins simultaneously in wide-field microscopy. Images are analysed using a classic autoencoder that identifies proteins with an accuracy of at least 80% (on the subset of proteins the authors tested).*

We thank the Reviewer for the comments.

*However, before recommending publication, the following major concerns should be addressed:*

*1- The introduction of five different epitopes might be expected to affect protein structure, function and organisation inside the cell. The authors refer to widefield images taken with the antibodies targeting the barcodes proteins which, in my opinion, do not confirm that the presence of all these epitopes on a target of interest do not change its structure, function and organisation. The authors are requested to validate their approach by carrying out functional assays as well as super-resolution/CryoEM imaging system on a randomly selected subset of proteins to proof that indeed the presence of these tags do not affect their targets. And, if the presence of the tags does affect the underlying proteins, what is the minimal number of tags that can be used for this approach to yield trustable result, does that minimum number of tags provide additional advantage over single colour/single protein imaging which allows up to 3/4 proteins to be simultaneously identified, and does reducing the number of tags reduce the accuracy (by how much) of the auto encoder in accurately identifying proteins.*

We agree with the Reviewer that this is an important issue. To reply thoroughly to this comment, we performed a series of experiments in which we tested all of the proteins that we used throughout the rest of this work.

However, some of our barcoded proteins do not actually have a cellular function. This subset includes cytosolic GFP, the nuclear localization signal (NLS), the ER-retention signal (KDEL sequence), and a mitochondria localization sequence (TOM70). To determine whether the epitopes alter the behavior of these proteins, we analyzed their colocalization to the compartments they should be present in, relying on two-color microscopy experiments (**Fig 1**, below). We also added GalNacT to this experiment, because its localization in the Golgi apparatus is essential for its function [1], and because functional assays for this protein are not easily implemented in microscopy experiments.

All other barcoded proteins are involved in membrane trafficking in the cell, which implies that they can be readily tested by classical assays designed to test receptor and cargo trafficking. We chose two such assays, which were performed in parallel.

First, we used a transferrin endocytosis and recycling assay. The protein transferrin is involved in iron metabolism in all mammalian cells, and is readily endocytosed, upon binding to its receptor. Transferrin is then recycled and released from the cells, within a time frame of a few tens of minutes [2]. This enables the microscopy investigation of both transferrin uptake during pulsing with fluorescently-conjugated transferrin, as a measure of endocytosis potential, and transferrin loss after a chase, as a measure of recycling and exocytosis.

Second, we relied on the endocytosis of the epidermal growth factor (EGF) receptor. The addition of fluorescently-conjugated EGF onto the cells results in abundant ligand-mediated endocytosis of the receptors, which are not recycled, but proceed slowly to the lysosomal compartment, where they are later degraded [3]. Therefore, no substantial loss of EGF fluorescence is expected upon a chase of a few tens of minutes, offering a different readout to transferrin.

We performed both of these assays for the endosomal membrane organizer Rab5a, for Lifeact (whose binding to actin should lead to a small, but measurable enhancement of actin dynamics, [4], and seven SNARE molecules involved in fusion events in the membrane trafficking pathway: endobrevin, syntaxins 4, 6, 7 and 13, Vti1a and VAMP4.

The expected result is that the overexpression of these proteins will not affect the transferrin and EGF dynamics negatively, but would rather lead to small enhancements of their uptake (and possibly release as well, for transferrin). The enhancements can only be limited, since the membrane trafficking pathways remain limited by the abundance of many other proteins, which are not overexpressed. We obtained this result for all proteins, as shown below. **Fig 2** presents an overall view of the results, indicating the transferrin and EGF dynamics in all experiments, combined. **Figs. 3** to **11** show the results for every individual protein, comparing the transferrin and EGF signals to the levels of overexpression of the respective proteins. Overall, these experiments indicate that these components of the membrane trafficking machinery are not negatively affected by our tagging procedure.

One additional SNARE molecule, SNAP25, is more difficult to test in such experiments, since it only functions in synapses, where its abundance is already extraordinary [5], so that overexpression is not expected to lead to changes in synaptic processes (just as lowering SNAP25 levels in heterozygous *SNAP25^+/-^* mice leads to very minor phenotypes, [6]. To validate the behavior of SNAP25, we therefore relied on a super-resolution imaging assay, in which we tested its localization, in comparison to endogenous SNAP25, in a neuroblastoma cell line (PC12). The results, shown in **Fig 12**, indicate that our epitope tagging does not affect SNAP25 localization. A quantification of localization results, also including the work relating to the proteins lacking a cellular function, is shown in **Fig 13**.

All of these figures have been added to the manuscript, as Suppl. Fig 5 to 17.

Finally, we would like to point out that the Reviewer is indeed right in surmising that some proteins will not be efficiently tagged using nanobarcodes. Tubulin is well-known for not allowing large tags, as they interfere with microtubule formation. Nanobarcoded tubulin was not functional (**Fig 14**), and was not used in our work, for any experiments, being discarded during the process of general testing for our experiments. This was the only protein where we discovered tag-related effects.

**Fig 1: Visualization of nanobarcoded proteins that act as markers for specific organelles**. The proteins indicated in the left-most column are markers for specific compartments, indicated in the next column. The colocalization of these proteins and specific compartment markers is then indicated in the fluorescence images. Scale bar: 20 µm. For quantification, see Fig 13, below.

**Fig 2. A functional assay to test nanobarcoded proteins.** Cells expressing different nanobarcoded proteins were pulsed with transferrin conjugated to Alexa488 and with EGF conjugated to Alexa647, for 10 minutes, allowing the cells to endocytose these ligands. Afterwards, they were immediately fixed, or were chased (washed off) in a minimal buffer at 37°C, for 10 or 20 minutes. Finally, all cells were fixed and immunolabeled for the ALFA tag, to identify the nanobarcoded proteins. (A-B) The behavior of transferrin and EGF, respectively. Transferrin recycles, as expected, being released during the chase period (Kruskal-Wallis test followed by Tukey post-hoc test, *p*<0.006 for endocytosis vs. 10- or 20-minute wash-off). No changes were seen for EGF, as expected (Kruskal-Wallis test, not significant). N = 17-18 independent experiments. (C-D) Same data as above, but indicating the nature of the nanobarcoded protein in each of the independent experiments.

8

**Fig 3. Transferrin and EGF imaging assays, tested for nanobarcoded Vti1a.** (A) Visualization of transferrin-Alexa488 (green) and EGF-Alexa647 (magenta), as well as the transfected protein, visualized with the ALFA nanobody (NbALFA) conjungated to AZdye568 (white). The three rows show the 10-minute pulse with the ligands (endocytosis), followed by the 10- and 20-minute chase (wash-off). To enable optimal visualization, the images are scaled differently, with the image scaling indicated in all panels. Scale bar: 20 µm. (B) The nanobarcoding scheme and the expected localization of the protein. (C) The NbALFA fluorescence intensity is plotted against the transferrin (green) and EGF (magenta) intensity, for all signals measured in two independent experiments, for all conditions. All intensities were normalized to the medians of the distributions, and were then grouped in 20 bins of ALFA intensity, each containing similar numbers of values. The mean and SEM of each bin, in the respective channels, is plotted. (D) The Pearson’s correlation coefficients for the distributions from panel C are shown, with the *p* values corrected for multiple testing using a Bonferroni correction.

**Fig 4. Transferrin and EGF imaging assays, tested for nanobarcoded syntaxin 4.** (A) Visualization of transferrin-Alexa488 (green) and EGF-Alexa647 (magenta), as well as the transfected protein, visualized with the ALFA nanobody (NbALFA) conjungated to AZdye568 (white). The three rows show the 10-minute pulse with the ligands (endocytosis), followed by the 10- and 20-minute chase (wash-off). To enable optimal visualization, the images are scaled differently, with the image scaling indicated in all panels. Scale bar: 20 µm. (B) The nanobarcoding scheme and the expected localization of the protein. (C) The NbALFA fluorescence intensity is plotted against the transferrin (green) and EGF (magenta) intensity, for all signals measured in two independent experiments, for all conditions. All intensities were normalized to the medians of the distributions, and were then grouped in 20 bins of ALFA intensity, each containing similar numbers of values. The mean and SEM of each bin, in the respective channels, is plotted. (D) The Pearson’s correlation coefficients for the distributions from panel C are shown, with the *p* values corrected for multiple testing using a Bonferroni correction.

**Fig 5. Transferrin and EGF imaging assays, tested for nanobarcoded syntaxin 6.** (A) Visualization of transferrin-Alexa488 (green) and EGF-Alexa647 (magenta), as well as the transfected protein, visualized with the ALFA nanobody (NbALFA) conjungated to AZdye568 (white). The three rows show the 10-minute pulse with the ligands (endocytosis), followed by the 10- and 20-minute chase (wash-off). To enable optimal visualization, the images are scaled differently, with the image scaling indicated in all panels. Scale bar: 20 µm. (B) The nanobarcoding scheme and the expected localization of the protein. (C) The NbALFA fluorescence intensity is plotted against the transferrin (green) and EGF (magenta) intensity, for all signals measured in two independent experiments, for all conditions. All intensities were normalized to the medians of the distributions, and were then grouped in 20 bins of ALFA intensity, each containing similar numbers of values. The mean and SEM of each bin, in the respective channels, is plotted. (D) The Pearson’s correlation coefficients for the distributions from panel C are shown, with the *p* values corrected for multiple testing using a Bonferroni correction.

**Fig 6. Transferrin and EGF imaging assays, tested for nanobarcoded syntaxin 7.** (A) Visualization of transferrin-Alexa488 (green) and EGF-Alexa647 (magenta), as well as the transfected protein, visualized with the ALFA nanobody (NbALFA) conjungated to AZdye568 (white). The three rows show the 10-minute pulse with the ligands (endocytosis), followed by the 10- and 20-minute chase (wash-off). To enable optimal visualization, the images are scaled differently, with the image scaling indicated in all panels. Scale bar: 20 µm. (B) The nanobarcoding scheme and the expected localization of the protein. (C) The NbALFA fluorescence intensity is plotted against the transferrin (green) and EGF (magenta) intensity, for all signals measured in two independent experiments, for all conditions. All intensities were normalized to the medians of the distributions, and were then grouped in 20 bins of ALFA intensity, each containing similar numbers of values. The mean and SEM of each bin, in the respective channels, is plotted. (D) The Pearson’s correlation coefficients for the distributions from panel C are shown, with the *p* values corrected for multiple testing using a Bonferroni correction.

**Fig 7. Transferrin and EGF imaging assays, tested for nanobarcoded endobrevin.** (A) Visualization of transferrin-Alexa488 (green) and EGF-Alexa647 (magenta), as well as the transfected protein, visualized with the ALFA nanobody (NbALFA) conjungated to AZdye568 (white). The three rows show the 10-minute pulse with the ligands (endocytosis), followed by the 10- and 20-minute chase (wash-off). To enable optimal visualization, the images are scaled differently, with the image scaling indicated in all panels. Scale bar: 20 µm. (B) The nanobarcoding scheme and the expected localization of the protein. (C) The NbALFA fluorescence intensity is plotted against the transferrin (green) and EGF (magenta) intensity, for all signals measured in two independent experiments, for all conditions. All intensities were normalized to the medians of the distributions, and were then grouped in 20 bins of ALFA intensity, each containing similar numbers of values. The mean and SEM of each bin, in the respective channels, is plotted. (D) The Pearson’s correlation coefficients for the distributions from panel C are shown, with the *p* values corrected for multiple testing using a Bonferroni correction.

**Fig 8. Transferrin and EGF imaging assays, tested for nanobarcoded LifeAct.** (A) Visualization of transferrin-Alexa488 (green) and EGF-Alexa647 (magenta), as well as the transfected protein, visualized with the ALFA nanobody (NbALFA) conjungated to AZdye568 (white). The three rows show the 10-minute pulse with the ligands (endocytosis), followed by the 10- and 20-minute chase (wash-off). To enable optimal visualization, the images are scaled differently, with the image scaling indicated in all panels. Scale bar: 20 µm. (B) The nanobarcoding scheme and the expected localization of the protein. (C) The NbALFA fluorescence intensity is plotted against the transferrin (green) and EGF (magenta) intensity, for all signals measured in two independent experiments, for all conditions. All intensities were normalized to the medians of the distributions, and were then grouped in 20 bins of ALFA intensity, each containing similar numbers of values. The mean and SEM of each bin, in the respective channels, is plotted. (D) The Pearson’s correlation coefficients for the distributions from panel C are shown, with the *p* values corrected for multiple testing using a Bonferroni correction.

**Fig 9. Transferrin and EGF imaging assays, tested for nanobarcoded Rab5a.** (A) Visualization of transferrin-Alexa488 (green) and EGF-Alexa647 (magenta), as well as the transfected protein, visualized with the ALFA nanobody (NbALFA) conjungated to AZdye568 (white). The three rows show the 10-minute pulse with the ligands (endocytosis), followed by the 10- and 20-minute chase (wash-off). To enable optimal visualization, the images are scaled differently, with the image scaling indicated in all panels. Scale bar: 20 µm. (B) The nanobarcoding scheme and the expected localization of the protein. (C) The NbALFA fluorescence intensity is plotted against the transferrin (green) and EGF (magenta) intensity, for all signals measured in two independent experiments, for all conditions. All intensities were normalized to the medians of the distributions, and were then grouped in 20 bins of ALFA intensity, each containing similar numbers of values. The mean and SEM of each bin, in the respective channels, is plotted. (D) The Pearson’s correlation coefficients for the distributions from panel C are shown, with the *p* values corrected for multiple testing using a Bonferroni correction.

**Fig 10. Transferrin and EGF imaging assays, tested for nanobarcoded syntaxin 13.** (A) Visualization of transferrin-Alexa488 (green) and EGF-Alexa647 (magenta), as well as the transfected protein, visualized with the ALFA nanobody (NbALFA) conjungated to AZdye568 (white). The three rows show the 10-minute pulse with the ligands (endocytosis), followed by the 10- and 20-minute chase (wash-off). To enable optimal visualization, the images are scaled differently, with the image scaling indicated in all panels. Scale bar: 20 µm. (B) The nanobarcoding scheme and the expected localization of the protein. (C) The NbALFA fluorescence intensity is plotted against the transferrin (green) and EGF (magenta) intensity, for all signals measured in two independent experiments, for all conditions. All intensities were normalized to the medians of the distributions, and were then grouped in 20 bins of ALFA intensity, each containing similar numbers of values. The mean and SEM of each bin, in the respective channels, is plotted. (D) The Pearson’s correlation coefficients for the distributions from panel C are shown, with the *p* values corrected for multiple testing using a Bonferroni correction.

**Fig 11. Transferrin and EGF imaging assays, tested for nanobarcoded VAMP4.** (A) Visualization of transferrin-Alexa488 (green) and EGF-Alexa647 (magenta), as well as the transfected protein, visualized with the ALFA nanobody (NbALFA) conjungated to AZdye568 (white). The three rows show the 10-minute pulse with the ligands (endocytosis), followed by the 10- and 20-minute chase (wash-off). To enable optimal visualization, the images are scaled differently, with the image scaling indicated in all panels. Scale bar: 20 µm. (B) The nanobarcoding scheme and the expected localization of the protein. (C) The NbALFA fluorescence intensity is plotted against the transferrin (green) and EGF (magenta) intensity, for all signals measured in two independent experiments, for all conditions. All intensities were normalized to the medians of the distributions, and were then grouped in 20 bins of ALFA intensity, each containing similar numbers of values. The mean and SEM of each bin, in the respective channels, is plotted. (D) The Pearson’s correlation coefficients for the distributions from panel C are shown, with the *p* values corrected for multiple testing using a Bonferroni correction.

**Fig 12: Endogenous SNAP25 and SNAP25(1100) have a similar cellular distribution within SNAP25(1100) transfected PC12 cells.** (A) Visualization of both endogenous SNAP25 and SNAP25(1100) using SNAP25 specific primary and secondary antibodies, plus NbALFA. (B-C) Negative control experiments, leaving out either primary antibodies (B) or NbALFA (C). (D) Imaging control, using a mixture of the same secondary antibody with two distinct fluorophores (targeting both endogenous SNAP25 and SNAP25(1100)), to provide a visual indication of the maximum expected colocalization. Bottom part of the Fig: legend for used symbols and schemes. Scale bar: 2.5 µm. For quantification, see Fig 13, below.

**Fig 13. An analysis of the colocalization of epitope-tagged proteins to their expected compartments.** The images from Fig 1 and 12 were analyzed by measuring the Pearson’s correlation coefficient in different image regions. The box plot indicates the respective values, compared to a control, consisting of similar measurements across the same regions in the protein-of-interest channel, and mirrored regions in the compartment channel. All proteins show a colocalization that is significantly above the control values (Kruskal-Wallis test followed by Tukey post-hoc test, *p*<0.006 for all proteins).

**Fig 14. Nanobarcoded tubulin does not function as endogenous tubulin.** Alpha-tubulin was tagged as indicated in the scheme, and was expressed in cultured HEK cells. Even when expressed at low levels, tagged alpha-tubulin diffuses in the whole cell, without assembling into microtobules, which indicates faulty function. Scale bar: 20 µm.

*2- It is not clear how can a pixel-wise autoencoder classify proteins imaged in epi (or even confocal) fluorescence where the axial excitation depth is large enough that many (many) proteins might emit onto the same pixel. This approach cannot replace the widely-used RGB colour format where a single pixel is represented by different colours representing the ratio of different proteins imaged on that pixel. The authors need to comment on that limitation and to inform the reader what kind of readout they should expect from this approach.*

The Reviewer is right about the relationship between axial excitation depth and protein emission onto the same pixel. Indeed, single pixels reflect the emission of many single proteins, and our analysis will only indicate the most common one present in the respective pixel. In our work, single pixels typically reflect only one type of nanobarcode, since the tagged molecules are expressed in different compartments, with limited overlap. To avoid this problem, one needs to increase the resolution of the microscopy technique used. In principle, our work has now reached the molecular resolution, in up to 4 channels [7], so that resolution would not be limiting for such approaches in the future.

In the revised manuscript, we now comment on this limitation (Discussion, p. 11, l. 258-2634).

*3- It is claimed that this approach can be easily extended to super-resolution microscopy, however, this seems to be non-trivial. The approach relies on 3/4 (spectrally-distinct) colour imaging. Multi-colour imaging using dSTORM has been successfully demonstrated using a maximum of 2 spectrally-distinct colours (e.g. 561 nm and 647 nm) given that 405 nm and 488 nm are used for activating fluorophores. Multi-colour imaging using spectrally-overlapping fluorophores was shown using prisms and diffraction-gratings - however, these approaches are not compatible with nanocoding (given that they rely on multiple distinct colours). Nanobarcoding can be combined with DNA-PAINT/STORM(not dSTORM) based imaging, but can the authors comment how this can be done and the limitations accrued from the merge and what can this approach deliver more compared to spectral-demixing approaches described in (https://doi.org/10.1038/nmeth.3528 or* [*https://doi.org/10.1101/2021.12.23.473862*](https://doi.org/10.1101/2021.12.23.473862) *or*
[*https://doi.org/10.1021%2Facs.nanolett.9b00508*](https://doi.org/10.1021%2Facs.nanolett.9b00508)*)*

We now mention this in the revised manuscript, including the Reviewer’s comments on DNA-PAINT/STORM and the potential benefits of nanobarcoding in comparison to spectral-demixing approaches (Discussion, p. 11-12, l. 264-276). In principle, all our nanobodies are readily available for DNA-PAINT approaches, and some have already been used for multiplexing [8], implying that a simple approach for nanobarcoding would be to rely on DNA-PAINT, imaging each nanobody sequentially. Alternatively, to maintain the ease of use of multicolor imaging experiments, we could rely on expansion microscopy, in which the sample is labeled with nanobodies, exactly as we now performed, and is then embedded in a swellable gel and is expanded [9]. This type of procedure could raise the resolution of the images by 5-10 fold, depending on the expansion factor of the gel, without major changes to our overall approach (Discussion, p. 12, l. 277-282).

*4- The imaging setup is not adequately described. The type of dichroic, emission and excitation filters is not described. It is not described whether the excitation is launched by fibre or free space launching. This information is important for others to replicate the imaging conditions - specially given that the entire method relies on the relative intensities of imaged colours.*

We added this information to the Methods section (p. 16, l. 377-380).

*5- The training of the NN is not well described either. The number of batches, epochs, etc. are not described and the NN (raw and trained) are not open-sourced or packaged. This tool, if appropritately validated as in 1, would need to be accessible to biologists for them to be able to replicate. The authors would also need to mention the time taken to train and deploy the NN and the computational environment used to achieve these.*

That has been an oversight on our part. We added text to the Methods section (p. 19-20, l. 457-467 & l. 476‑482) to provide the requested information. Also, the software package developed for training and application of the neural net is now available publicly via <https://github.com/noegroup/deep_nanobarcode>. Network parameters, weights, training data, and images used for training and testing the network have all been made publicly available via an ftp server and data hosted on Refubium repository of Freie Universität Berlin. We have explained all the details in the Software and Data Availability Paragraph. We also added text to highlight the computational environment (PyTorch), the hardware used (GeForce RTX 3090 graphics card), and the time needed for training and inference with the neural network.

***Reviewer #2:*** *The authors set out the potential for microscopy in performing highly multiplexed imaging to determine the spatial organisation of multiple markers at one time, yet only a handful of channels being practically tractable. They present an approach for solving this problem by using combinatorial epitope labelling. A series of constructs are generated with different assortments of epitope tags including the ALFAtag, GFP and mCherry which are then detected using fluorophore-conjugated nanobodies in fixed cells, termed 'nanobarcoding'. The samples are excited with each of 4 wavelengths in turn and the pixel intensity values are determined across the series of images at with each set of emission filters. The per-pixel intensity values are then used to train an neural network to identify which "nanobarcode" is present. I agree with the authors about the importance of the barrier and that combinatorial tagging is an attractive approach.*

We thank the Reviewer for the comments.

*My main comment is that the manuscript as prepared isn't giving me all the information I need to evaluate the method in a clear enough manner. The text is overly abbreviated, including the abstract. To give an example, on page 4 the sentence "we verified the accuracy (Suppl. Fig 3-4)... appeared to function well" is to capture 5 pages of data on the foundation of the method.*

We agree with the reviewer about the manuscript text being too brief at points. We tried to add the necessary information for all the points mentioned by the Reviewer.

*For a methods paper I am expecting to see the choice of controls clearly explained, performed and stated, such as for antibody specificity and whether the conjugated proteins are being accurately expressed and to what levels (e.g. an assessment by western blotting as well as the images).*

We agree with the Reviewer that this is a very important issue. To reply thoroughly to this comment, we performed a series of experiments in which we tested all of the proteins that we used throughout the rest of this work. As Reviewer #1 also asked about this point, we introduced all of these experiments above.

First, some of our barcoded proteins (GFP, the nuclear localization signal, NLS, the ER-retention signal, KDEL sequence, and a mitochondria localization sequence, TOM70) do not actually have a cellular function, but act as markers of specific compartments, we analyzed their colocalization to these compartments, relying on two-color microscopy experiments (**Fig 1**, above).

Second, all other barcoded proteins are involved in membrane trafficking in the cell, which implies that they can be readily tested by classical assays designed to test receptor and cargo trafficking. We chose two such assays, which were performed in parallel. First, we used a transferrin endocytosis and recycling assay. The protein transferrin is involved in iron metabolism in all mammalian cells, and is readily endocytosed, upon binding to its receptor. Transferrin is then recycled and released from the cells, within a time frame of a few tens of minutes [2]. This enables the microscopy investigation of both transferrin uptake during pulsing with fluorescently-conjugated transferrin, as a measure of endocytosis potential, and transferrin loss after a chase, as a measure of recycling and exocytosis. Second, we relied on the endocytosis of the epidermal growth factor (EGF) receptor. The addition of fluorescently-conjugated EGF onto the cells results in abundant ligand-mediated endocytosis of the receptors, which are not recycled, but proceed slowly to the lysosomal compartment, where they are later degraded [3]. Therefore, no substantial loss of EGF fluorescence is expected upon a chase of a few tens of minutes, offering a different readout to transferrin. The expected result is that the overexpression of these proteins will not affect the transferrin and EGF dynamics negatively, but would rather lead to small enhancements of their uptake (and possibly release as well, for transferrin). The enhancements can only be limited, since the membrane trafficking pathways remain limited by the abundance of many other proteins, which are not overexpressed. We obtained this result for all proteins. **Fig 2**, above, presents an overall view of the results, indicating the transferrin and EGF dynamics in all experiments, combined. **Figs 3** to **11** show the results for every individual protein, comparing the transferrin and EGF signals to the levels of overexpression of the respective proteins. Overall, these experiments indicate that these components of the membrane trafficking machinery are not negatively affected by our tagging procedure.

One additional SNARE molecule, SNAP25, was more difficult to test in such experiments, since it only functions in synapses, where its abundance is already extraordinary [5], so that overexpression is not expected to lead to changes in synaptic processes (just as lowering SNAP25 levels in heterozygous *SNAP25^+/-^* mice leads to very minor phenotypes, [6]. To validate the behavior of SNAP25, we therefore relied on a super-resolution imaging assay, in which we tested its localization, in comparison to endogenous SNAP25, in a neuroblastoma cell line (PC12). The results, shown in **Fig 12**, indicate that our epitope tagging does not affect SNAP25 localization. A quantification of localization results, also including the work relating to the proteins lacking a cellular function, is shown in **Fig 13**.

All of these figures have been added to the manuscript, as Suppl. Figs 5 to 17.

*Sometimes supplementary figures are referred to in advance of the main figures (e.g Supp Figs 6 and 7, page 5) and even the supplementary figures are not giving me the levels of information and explanation I need to properly assess the approach. It is also not clear whether each user would need to train and create their own neural network or if there is analysis software the authors developed that can be straightforwardly implemented by potential users (probably not if it is not obvious, which limits the generality of the approach).*

We added more details about the training and application of the deep network. We also made the software package open source and publicly available via the github repository <https://github.com/noegroup/deep_nanobarcode>. All the data that the end-user needs to test the network on their own machine has been made documented and made publicly available. The trained network should be transferable to new data, within the limit of instrumental differences. Further training of the network is also possible without the need to modify the code. We have provided our training data, plus the code needed for retraining/fine-tuning the network.

*In terms of the science, the nanobarcoding tags are very large. I question the choice of GFP and mCherry rather than smaller epitope tags. For presenting a method I would expect to see this choice evaluated and compared with other possible choices.*

At the time we started our initial experiments, there were only a handful of nanobodies available, which limited our flexibility. We see the benefits of smaller epitope tags, yet Fluorescent (E)GFP and mCherry are still commonly used tags, which have been extensively validated in the literature. The exchange of one amino acid, resulting in the loss of fluorescence, as used for our constructs, is unlikely to influence the overall structure and function of the proteins. Indeed, our complementary validation experiments indicate there are no major restrictions in structure or function of nanobarcoded proteins (independent of the epitopes present), as described above (Figs. 1 to 13).

In the revised manuscript we have also added the following table, giving an overview of validation experiments which have been already published and/or have been performed for the revision of this manuscript (Table 1, below).

The table has been added to the manuscript, as Suppl. Table 1

| **Protein** | **Validation in literature** | **Validation in this manuscript** |
| --- | --- | --- |
| 1. Vti1a(0001) | - [10–12] | EGF and Transferrin experiments  (Suppl. Fig 7) |
| 2. Syntaxin4(0010) | - [13,14] - Synuclein Nanobody epitopes / tags are short [15–17] | EGF and Transferrin experiments  (Suppl. Fig 8) |
| 3. Syntaxin6(0011) | - [10] - Synuclein Nanobody epitopes / tags are short [15–17] | EGF and Transferrin experiments (Suppl. Fig 9) |
| 4. GFP(0100) | - No known function, therefore no functional validation needed; only verification of localization needed. | Colocalization assays  (Suppl. Fig 5) |
| 5. STX7(0101) | - [18–20] and probably others as well | EGF and Transferrin experiments  (Suppl. Fig 10) |
| 6. GalNacT(0110) | - Localization is related to fuction, so was investigated here [1]. | Colocalization assays  (Suppl. Fig 5) |
| 1. 2. 3. 4. 5. 6. 7. Endobrevin(0111) | - [21,22] | EGF and Transferrin experiments  (Suppl. Fig 11) |
| 8. TOM70(1000) | - No known function, therefore no functional validation needed; only verification of localization needed. | Colocalization assays  (Suppl. Fig 5) |
| 9. LifeAct(1001) | - Actin-binding molecule, designed to be tagged [23]. | EGF and Transferrin experiments  (Suppl. Fig 12) |
| 10. Rab5a(1010) | - [10,11,24,25] | EGF and Transferrin experiments  (Suppl. Fig 13) |
| 11. STX13(1011) |  | EGF and Transferrin experiments  (Suppl. Fig 14) |
| 12. SNAP25(1100) | - Tested in cell lines [10,26–28] | STED SNAP25 (PC 12 cells)  (Suppl. Fig 16) |
| 13. NLS(1101) | - No known function, therefore no functional validation needed; only verification of localization needed. | Colocalization assays  (Suppl. Fig 5) |
| 14. KDEL(1110) | - No known function, therefore no functional validation needed; only verification of localization needed. | Colocalization assays  (Suppl. Fig 5) |
| 15. VAMP4(1111) | - [10,29,30] | EGF and Transferrin experiments  (Suppl. Fig 15) |

Table 1: Protein tag validation in literature and/or in this manuscript.

*The biological example of Nrxns and Nlgns used to illustrate the technique is not sufficiently well explained for the wide readership of a methods paper.*

We have added more explanations to this section.

*The false positive and false negative rates are substantial, typically >10% which significantly limits the utility of the approach in my view.*

Our reported false positive/negative percentage values seem to have been misleading. The values we presented have been percentages calculated from all false predictions, and not absolute percentages from all predictions, as desired by the Reviewer. We have fixed this by reporting values in absolute percentages, thereby showing substantially smaller values (<4%). We additionally report precision, recall and F1-scores (Fig 2, panels c &d), resulting from application of the deep network on hold-out test data.

*The authors discuss that the approach relies on exogenous expression and is the tags are currently too big for endogenous labelling.*

We are sorry for this misunderstanding. The tags are not too big for endogenous labeling. With the appropriate effort, one could knock-in these proteins, using modern genetics tools, and then use all molecules in an endogenous-like fashion.

*My view is that the size of the nanobarcodes is a significant limitation such that this technique is not really widely applicable and is actually ends up quite a niche approach, even if it is well implemented. At the same time I can't properly tell if it is successful/well implemented because the manuscript seems to be making too many assumptions about what their readers can discern without explanation.*

We apologize for this problem. We hope that the new version of the manuscript is clearer and more easily understandable.

*Reviewer #3: In this paper, D. Jong-Bolm et al. present a novel approach for multiplexed fluorescence microscopy. This involves the use of nanobodies combined with a deep learning analysis method. Using protein engineering, they could generate proteins containing 5 genetically encoded epitopes that are recognized by nanobodies. They designed a proof of principle study on 15 different proteins using 4 fluorescent tags. Confocal microscopy was performed with a 10 fluorescent channel recording scheme and a custom-based deep learning analysis was designed. The proposed method as the potential to have a strong impact in the field of multi-color imaging and could be combined with CRISPR/Cas gene editing technologies to extend its application to endogenous proteins. The validation experiments for the nanobody-based barcoding strategy are robust and convincing.*

We thank the Reviewer for the comments.

*While the analysis approach using deep learning is very promising, I think that essential validation steps are missing and should be included in the manuscript before publication.*

Please see our replies to the specific comments, below.

*1.      Training procedure of the neural network: in the Materials and Methods section, lines 248-251, the authors describe how the input data was split into training and hold-out test datasets. However, the training performance was monitored by comparing the performance on the training and testing dataset. From this description it appears to me that the testing set was used as a validation set and that no "independent test dataset" was used to report the performance. Therefore, we lack information on the generalization capacities of the proposed approach. The authors should show that the method can be trained on a dataset (split in test and validation sets) and evaluated on a completely independent testing dataset (never seen by the network during training and not used to monitor training performance during training).*

We had used the hold-out test set, to which the network was not exposed during training. But we agree that not clearly separating the validation and test data was an oversight. We have now split our data into 80%-10%-10% train/validation/test sets. All the metrics reported in the revised version correspond to the test set (not previously seen by the network, revised Fig 2. C and D, Supplementary Fig 19), while the hyperparameter-optimization and monitoring of the training procedure + early stopping is done based on the validation set (Methods and **Fig 16**).

The revised metrics are also shown below in **Fig 15 and 16**. In the revised manuscript, Fig 15 and Fig 16 are the new Supplementary Fig 19 and Fig 3, respectively.

**Fig 15. Deep network performance metrics for different protein expression times:** prediction accuracies (top panel titles), as well as precision, recall, and F1-Score (shown in the same order for each protein in the top panels), false positives and false negatives (bottom panels). Data are shown for overnight, 24 hrs and 72 hrs protein expression, respectively.

**Fig 16. Training and testing of the deep network**. (A) Pipeline through which data are prepared for training and testing the deep network for SNAP25 from 48 h protocol as an example. 10-dimensional vectors containing pixel-wise intensities across all channels are mapped along one dimension using kPCA transform. A relative threshold on the principal component separates foreground from background and results in a binary mask, based on which data can be gathered from points than contain proteins in the confocal image. (B) The result of Isomap, kPCA, t-SNE and Sepctral Embedding “shallow-learning” methods for dimensionality reduction applied directly to the data gathered according to the pipeline explained in (A). (C) Training and validation accuracies averaged over all proteins in the dataset, sampled in each training epoch. Red dashed line shows the early stopping used based on the monitored validation accuracy. (D) Results of the ablation study, in which in each case one protein is removed from the training dataset and the performance of the deep network is evaluated based on the given metrics after training and validation procedure is performed.

*2.      Datasets: A description of the dataset is lacking. This description should include at least: the number of images for each protein, the number of images for each time period (o/n, 24h, 48h, 72h), the size of the images (in microns and number of pixels), the size of training crops (if used). In the material and methods section, it should be specified if the reported performance (in Fig. 2) was obtained after training on the full dataset, or only on the dataset including the subset of proteins that are shown in Fig.2. It should also be mentioned if all images (for different expression time periods) were included in the training set.*

We have now included a more detailed description of the data pipeline used for training and testing the network in the Methods section “*Training and testing the deep network”*(p. 19-20, l. 456-482)*,* in **Fig 16**, above. and in **Table 2**, below. Table 2 is added as Suppl. Table 3 to the revised manuscript.

|  | **Protein** | **Time window** | **Number of Images** | **Total number of sampled pixels** |
| --- | --- | --- | --- | --- |
| 01 | Blank (no protein) | N/A | 24 | 2400000 |
| 02 | Endobrevin (0111) | overnight | 10 | 10000 |
|  |  | 24h | 12 | 10000 |
|  |  | 48h | 10 | 10000 |
|  |  | 72h | 5 | 10000 |
| 03 | GalNacT (0110) | overnight | 9 | 3224 |
|  |  | 24h | 12 | 10000 |
|  |  | 48h | 10 | 2340 |
|  |  | 72h | 5 | 10000 |
| 04 | GFP (0100) | overnight | 10 | 10000 |
|  |  | 24h | 12 | 10000 |
|  |  | 48h | 5 | 10000 |
|  |  | 72h | 5 | 10000 |
| 05 | KDEL (1110) | overnight | 5 | 10000 |
|  |  | 24h | 12 | 10000 |
|  |  | 48h | 10 | 10000 |
|  |  | 72h | 5 | 10000 |
| 06 | Lifeact (1001) | overnight | 5 | 10000 |
|  |  | 24h | 12 | 10000 |
|  |  | 48h | 10 | 10000 |
|  |  | 72h | 5 | 10000 |
| 07 | NLS (1101) | overnight | 10 | 10000 |
|  |  | 24h | 12 | 10000 |
|  |  | 48h | 10 | 10000 |
|  |  | 72h | 5 | 10000 |
| 08 | SNAP25 (1100) | overnight | 5 | 5412 |
|  |  | 24h | 12 | 10000 |
|  |  | 48h | 10 | 10000 |
|  |  | 72h | 5 | 10000 |
| 09 | STX4 (0010) | overnight | 10 | 10000 |
|  |  | 24h | 12 | 10000 |
|  |  | 48h | 10 | 10000 |
|  |  | 72h | 5 | 10000 |
| 10 | STX6 (0011) | overnight | 10 | 10000 |
|  |  | 24h | 12 | 10000 |
|  |  | 48h | 11 | 10000 |
|  |  | 72h | 5 | 10000 |
| 11 | TOM70 (1000) | overnight | 9 | 10000 |
|  |  | 24h | 7 | 10000 |
|  |  | 48h | 10 | 10000 |
|  |  | 72h | 5 | 1000 |
| 12 | Vti1a (1000) | overnight | 9 | 1033 |
|  |  | 24h | 12 | 10000 |
|  |  | 48h | 10 | 10000 |
|  |  | 72h | 5 | 3825 |

**Table 2. Summary of the image data used for training and testing the deep network.** Number of confocal images obtained for each protein (with the given nanobarcode) as well as number of pixels that have been sampled for the deep learning dataset. All images have the same dimensions of 512x512 pixels. With 72 h samples, each image contains 5 slices in a z-stack. Network training, validation, and testing are done only based on the sub-sampled pixels (with the numbers given in the last column), while the precision matrices in Supplementary Fig 20 are obtained on full-frame images.

*3.      Prediction accuracy: The prediction accuracy of the network is provided only for a subset of proteins in Fig.2C&D, however it is not clear how it compares with the performance on the other proteins. A careful description of the performance on all 15 proteins should be included as well as an ablation study showing how reducing/increasing the number of detected protein targets affect the prediction accuracy. Results of the deep learning approach should be compared to established hyperspectral imaging baselines.*

We now report the prediction precision, recall, and F1-score for all proteins in the training/test datasets. We have also included an ablation study, in which the sensitivity of the network to each protein is measure **Fig 16D**, above. To compare our deep learning approach with other possible clustering/dimensionality reduction techniques applied on the hyperspectral data, we included results of Isomap, kPCA, t-SNE and Spectral Embedding analyses. None of these methods came close to the possibility of successfully classifying the imaged nanobarcodes.

*4.      In supplementary Fig. 6, the Neural network analysis results are presented, and the accuracy seems very low (below 0.4) for multiple proteins and multiple training/testing combinations. The authors should discuss these results in the manuscript and compare the results to the one obtained with established hyperspectral imaging baselines. The approach used to generate the results in supplementary fig. 6 should be described in the materials and methods section.*

In this revision, we made significant improvement to the deep learning procedure. We implemented a new data pipeline for gathering higher quality training data (explained in details the Methods section). We also changed network hyperparameters, and made the network larger. With these modifications, we achieved much better prediction precision with the network and have included the new results instead.

*5.      It is not clear to me why the validation images were taken on a different microscope and if those images were used to validate the deep learning approach. On line 86 they mention that the performance was analyzed on the hold-out test sets and on full images of single-transfected samples. Are those images of single-transfected samples the same as the validation images described at line 206? If so, the authors should specify how they have checked the generalizability of the approach on different microscopes.*

We performed different validation experiments for the proteins using several microscopes, which are more adapted to the respective experiments. These experiments were only implemented to determine whether the proteins behaved as expected, and could be used in multicolor imaging and the subsequent deep learning approaches. All work relating to the deep network was performed on one confocal microscope.

The deep network developed in our work should be flexible enough to be retrained/fine-tuned with new training data obtained from another microscope. Nevertheless, we cannot claim that we can cover all imaging conditions and instrument variations in one set of weights (network). Therefore, we have also provided the possibility of retraining the network in the software package (https://github.com/noegroup/deep_nanobarcode).

*6.      Data availability statement: In the manuscript the data availability statement states that "The data presented in this study are available on request to the corresponding author", while in the section Data Availability found in the table preceding the manuscript it is stated that all data are fully available without restriction. Considering that this manuscript presents results on image analysis with deep learning, it is essential that the authors provide a curated and open-source dataset as well as a fully documented and open-source code.*

We agree with the Reviewer that the data availability should have been more transparent. We have extended our Software and Data Availability paragraph. In fact, all the images used for training and testing the deep network, as well as the network’s parameters and saved weights, are now available via public repositories. Optimized network hyperparameters, weights of the trained networks and training datasets are all freely available for download from the ftp server <ftp://ftp.mi.fu-berlin.de/pub/cmb-data/deep_nanobarcode>. The software package additionally includes functionality for automatically downloading all the needed data. Images used for training and testing the deep network are available in the original format (LSM) from a Refubium repository hosted by the Free University of Berlin and accessible via the link <http://dx.doi.org/10.17169/refubium-39512>.

*7.      Reproducibility of the approach: The information on the deep learning-based image analysis procedure is insufficient to reproduce the analysis presented in this manuscript. The author should provide access to a fully operational analysis code, including the default configurations with optimized hyperparameters and images to test the approach. A more detailed description of the optimized hyperparameters should be also provided in the method section.*

We agree with the reviewer on the need for an open-source software package. Our software Deep-Nanobarcode is publicly available with an MIT license via the repository at <https://github.com/noegroup/deep_nanobarcode>. We have also included more details on the network configuration and hyperparameters in the Methods section.

*Overall, if the above-mentioned major comments are addressed by the authors, I would recommend this paper for publication given that the deep learning approach shows robust and generalizable results on an independent test set. I believe that this study can have a strong impact in the fields of multiplexed fluorescence microscopy and cell biology.*

We thank the Reviewer for the comments.

1.	Becker JL, Tran DT, Tabak LA. Members of the GalNAc-T family of enzymes utilize distinct Golgi localization mechanisms. Glycobiology. 2018;28: 841. doi:10.1093/GLYCOB/CWY071

2.	Schneider C, Williams JG. Molecular dissection of the human transferrin receptor. J Cell Sci Suppl. 1985;3: 139–149. doi:10.1242/JCS.1985.SUPPLEMENT_3.14

3.	Alexander A. Endocytosis and intracellular sorting of receptor tyrosine kinases. Front Biosci. 1998;3. doi:10.2741/A316

4.	Sliogeryte K, Thorpe SD, Wang Z, Thompson CL, Gavara N, Knight MM. Differential effects of LifeAct-GFP and actin-GFP on cell mechanics assessed using micropipette aspiration. J Biomech. 2016;49: 310–317. doi:10.1016/J.JBIOMECH.2015.12.034

5.	Wilhelm BG, Mandad S, Truckenbrodt S, Kroḧnert K, Schäfer C, Rammner B, et al. Composition of isolated synaptic boutons reveals the amounts of vesicle trafficking proteins. Science (80- ). 2014;344: 1023–1028. doi:10.1126/SCIENCE.1252884/SUPPL_FILE/WILHELM.SM.PDF

6.	Antonucci F, Corradini I, Fossati G, Tomasoni R, Menna E, Matteoli M. SNAP-25, a Known Presynaptic Protein with Emerging Postsynaptic Functions. Front Synaptic Neurosci. 2016;8. doi:10.3389/FNSYN.2016.00007

7.	Shaib AH, Chouaib AA, Chowdhury R, Mihaylov D, Zhang C, Imani V, et al. Visualizing proteins by expansion microscopy. bioRxiv. 2023; 2022.08.03.502284. doi:10.1101/2022.08.03.502284

8.	Sograte-Idrissi S, Oleksiievets N, Isbaner S, Eggert-Martinez M, Enderlein J, Tsukanov R, et al. Nanobody detection of standard fluorescent proteins enables multi-target DNA-PAINT with high resolution and minimal displacement errors. Cells. 2019;8. doi:10.3390/cells8010048

9.	Chen F, Tillberg PW, Boyden ES. Optical imaging. Expansion microscopy. Science. 2015;347: 543–548. doi:10.1126/SCIENCE.1260088

10.	Reshetniak S, Ußling J, Perego E, Rammner B, Schikorski T, Fornasiero EF, et al. A comparative analysis of the mobility of 45 proteins in the synaptic bouton. EMBO J. 2020;39. doi:10.15252/EMBJ.2020104596

11.	Hoopmann P, Punge A, Barysch S V., Westphal V, Bückers J, Opazo F, et al. Endosomal sorting of readily releasable synaptic vesicles. Proc Natl Acad Sci U S A. 2010;107: 19055–19060. doi:10.1073/PNAS.1007037107/-/DCSUPPLEMENTAL/SM01.MOV

12.	Ramirez DMO, Khvotchev M, Trauterman B, Kavalali ET. Vti1a identifies a vesicle pool that preferentially recycles at rest and maintains spontaneous neurotransmission. Neuron. 2012;73: 121–134. doi:10.1016/J.NEURON.2011.10.034

13.	Kalwat MA, Wiseman DA, Luo W, Wang Z, Thurmond DC. Gelsolin Associates with the N Terminus of Syntaxin 4 to Regulate Insulin Granule Exocytosis. Mol Endocrinol. 2012;26: 128–141. doi:10.1210/ME.2011-1112

14.	Takuma T, Arakawa T, Okayama M, Mizoguchi I, Tanimura A, Tajima Y. Trafficking of Green Fluorescent Protein-Tagged SNARE Proteins in HSY Cells. J Biochem. 2002;132: 729–735. doi:10.1093/OXFORDJOURNALS.JBCHEM.A003280

15.	Guilliams T, El-Turk F, Buell AK, O’Day EM, Aprile FA, Esbjörner EK, et al. Nanobodies Raised against Monomeric α-Synuclein Distinguish between Fibrils at Different Maturation Stages. J Mol Biol. 2013;425: 2397–2411. doi:10.1016/J.JMB.2013.01.040

16.	De Genst EJ, Guilliams T, Wellens J, Day EM, Waudby CA, Meehan S, et al. Structure and properties of a complex of α-synuclein and a single-domain camelid antibody. J Mol Biol. 2010;402: 326–343. doi:10.1016/J.JMB.2010.07.001

17.	El-Turk F, Newby FN, De Genst E, Guilliams T, Sprules T, Mittermaier A, et al. Structural Effects of Two Camelid Nanobodies Directed to Distinct C-Terminal Epitopes on α-Synuclein. Biochemistry. 2016;55: 3116–3122. doi:10.1021/ACS.BIOCHEM.6B00149

18.	Prekeris R, Yang B, Oorschot V, Klumperman J, Scheller RH. Differential roles of syntaxin 7 and syntaxin 8 in endosomal trafficking. Mol Biol Cell. 1999;10: 3891–3908. doi:10.1091/MBC.10.11.3891

19.	Mashima H, Suzuki J, Hirayama T, Yoshikumi Y, Ohno H, Ohnishi H, et al. Involvement of vesicle-associated membrane protein 7 in human gastric epithelial cell vacuolation induced by Helicobacter pylori-produced VacA. Infect Immun. 2008;76: 2296–2303. doi:10.1128/IAI.01573-07

20.	Mori Y, Takenaka K ichiro, Fukazawa Y, Takamori S. The endosomal Q-SNARE, Syntaxin 7, defines a rapidly replenishing synaptic vesicle recycling pool in hippocampal neurons. Commun Biol 2021 41. 2021;4: 1–13. doi:10.1038/s42003-021-02512-4

21.	Nagamatsu S, Nakamichi Y, Watanabe T, Matsushima S, Yamaguchi S, Ni J, et al. Localization of cellubrevin-related peptide, endobrevin, in the early endosome in pancreatic β cells and its physiological function in exo-endocytosis of secretory granules. J Cell Sci. 2001;114: 219–227. doi:10.1242/JCS.114.1.219

22.	Okayama M, Arakawa T, Tanimura A, Mizoguchi I, Tajima Y, Takuma T. Role of VAMP8/endobrevin in Constitutive Exocytotic Pathway in HeLa Cells. Cell Struct Funct. 2009;34: 115–125. doi:10.1247/CSF.09013

23.	Riedl J, Crevenna AH, Kessenbrock K, Yu JH, Neukirchen D, Bista M, et al. Lifeact: a versatile marker to visualize F-actin. Nat Methods. 2008;5: 605–607. doi:10.1038/NMETH.1220

24.	Bethani I, Lang T, Geumann U, Sieber JJ, Jahn R, Rizzoli SO. The specificity of SNARE pairing in biological membranes is mediated by both proof-reading and spatial segregation. EMBO J. 2007;26: 3981–3992. doi:10.1038/SJ.EMBOJ.7601820

25.	Pavlos NJ, Grønborg M, Riedel D, Chua JJE, Boyken J, Kloepper TH, et al. Quantitative analysis of synaptic vesicle Rabs uncovers distinct yet overlapping roles for Rab3a and Rab27b in Ca2+-triggered exocytosis. J Neurosci. 2010;30: 13441–13453. doi:10.1523/JNEUROSCI.0907-10.2010

26.	An SJ, Almers W. Tracking SNARE complex formation in live endocrine cells. Science. 2004;306: 1042–1046. doi:10.1126/SCIENCE.1102559

27.	Halemani ND, Bethani I, Rizzoli SO, Lang T. Structure and dynamics of a two-helix SNARE complex in live cells. Traffic. 2010;11: 394–404. doi:10.1111/J.1600-0854.2009.01020.X

28.	Rickman C, Medine CN, Dun AR, Moulton DJ, Mandula O, Halemani ND, et al. t-SNARE protein conformations patterned by the lipid microenvironment. J Biol Chem. 2010;285: 13535–13541. doi:10.1074/JBC.M109.091058

29.	Nicholson-Fish JC, Kokotos AC, Gillingwater TH, Smillie KJ, Cousin MA. VAMP4 Is an Essential Cargo Molecule for Activity-Dependent Bulk Endocytosis. Neuron. 2015;88: 973–984. doi:10.1016/J.NEURON.2015.10.043

30.	Tran THT, Zeng Q, Hong W. VAMP4 cycles from the cell surface to the trans-Golgi network via sorting and recycling endosomes. J Cell Sci. 2007;120: 1028–1041. doi:10.1242/jcs.03387
